# Joint Mapping and Allele Mining of the Rolled Leaf Trait in Rice (*Oryza sativa* L.)

**DOI:** 10.1371/journal.pone.0158246

**Published:** 2016-07-21

**Authors:** Qiang Zhang, Tianqing Zheng, Long Hoang, Chunchao Wang, Nafisah, Charles Joseph, Wenzhong Zhang, Jianlong Xu, Zhikang Li

**Affiliations:** 1 Shenyang Agricultural University, 120 Dongling Road, Shenyang 110866, China; 2 Institute of Crop Sciences/National Key Facility for Crop Gene Resources and Genetic Improvement, Chinese Academy of Agricultural Sciences, 12 South Zhong-Guan-Cun Street, Beijing 100081, China; 3 Agricultural Genomics Institute at Shenzhen, Chinese Academy of Agricultural Sciences, Shenzhen 518120, China; 4 Shenzhen Institute of Breeding & Innovation, Chinese Academy of Agricultural Sciences, Shenzhen 518120, China; University of Guelph, CANADA

## Abstract

The rolled leaf trait, long considered to be a key component of plant architecture, represents an important target trait for improving plant architecture at the population level. We therefore performed linkage mapping using a set of 262 highly variable RILs from two rice cultivars (Minghui 63 and 02428) with minor differences in leaf rolling index (LRI) in conjunction with GWAS mapping of a random subset of the 1127 germplasms from the 3K Rice Genomes Project (3K Rice). A total of seven main-effect loci were found to underlie the transgressive segregation of progenies from parents with minor differences in LRI. Five of these loci were previously identified and two (*qRl7b* and *qRl9b*) are newly reported with additional evidence from GWAS mapping for *qRl7b*. A total of 18 QTLs were identified by GWAS, including four newly identified QTLs. Six QTLs were confirmed by linkage mapping with the above RIL population, and 83.3% were found to be consistent with previously reported loci based on comparative mapping. We also performed allele mining with representative SNPs and identified the elite germplasms for the improvement of rolled leaf trait. Most favorable alleles at the detected loci were contributed by various 3K Rice germplasms. By a re-scanning of the candidate region with more saturated SNP markers, we dissected the region harboring *gRl4-2* into three subregions, in which the average effect on LRI was 3.5% with a range from 2.4 to 4.1% in the third subregion, suggesting the presence of a new locus or loci within this region. The representative SNPs for favorable alleles in the reliable QTLs which were consistently identified in both bi-parental mapping and GWAS, such as *qRl4*, *qRl5*, *qRl6*, *qRl7a*, and *qRl7b* will be useful for future molecular breeding programs for ideal plant type in rice.

## Introduction

The rolled (V-shaped or curled) leaf trait has long been considered by experienced breeders to be a key trait for ideal plant type not only for *indica* hybrid rice breeding [1] but also for inbred *japonica* cultivar development in Northern China [2]. Extremely rolled leaves often lead to reduced rates of photosynthesis and apoplastic transport ability [3] and even reduced light use efficiency [4]. At the individual level, rolled leaves are not always directly associated with yield component traits in certain crosses, such as MH63 × 02428 [5], let alone the unfavorable traits including dwarf and/or narrow leaves and/or smaller panicles often occur in conjunction with rolled leaves in most artificial mutants, except for a few natural mutants such as *rl(t)* [6]. Nonetheless, moderately rolled leaves can improve photosynthetic efficiency in certain cultivars [7,8] and thus contribute to economic and grain quality traits [6,9]. In addition, the rolled leaf trait is thought to contribute to lodging resistance and ventilation, which are strongly associated with disease resistance, especially to fungal diseases, at the population level [10]. Moreover, cultivars with moderately rolled leaves are suitable for cultivation at relatively high density [11].

To date, no fewer than 70 genes/QTLs for the rolled leaf trait have been mapped or cloned throughout the genome. Most studies of the rolled leaf trait have involved the use of parents with significant phenotypic differences. Unlike the extremely rolled leaf phenotype, moderately rolled leaves or leaves with various degrees of rolling, especially inside-rolled (adaxial rolled) leaves, would be a useful trait to target in breeding. Uncovering hidden diversity in the progenies of parents with minor phenotypic differences in target traits is also important for dissecting complex traits including resistance to various biotic and abiotic stresses [12, 13]. Whether the mechanisms underlying hidden diversity also function for relatively simple traits such as rolled leaves remains unclear.

Currently, mining favorable alleles is an important component of plant breeding [14]. However, traditional QTL detection by linkage mapping is usually performed using populations with limited parental variation [15]. Performing GWAS offers opportunities to overcome this shortcoming. If GWAS is performed jointly with linkage mapping, the relatively high false positive rate of GWAS is largely constrained, and the efficiency of QTL mapping is much improved, as demonstrated in maize [16]. This technique has been successfully employed in rice to help dissect complex biotic stress traits, such as rice black-streaked dwarf disease resistance, as well as relatively simple genetic traits including rice leaf stripe disease resistance [17]. The simultaneous exploration of natural variations would be highly useful for rice breeding.

Here, we utilized a traditional recombinant inbred line (RIL) population derived from two parents with minor differences in leaf rolling index (LRI) for linkage mapping, along with a germplasm panel from the 3K Rice [18], for joint mapping of loci affecting the rolled leaf trait and for mining favorable alleles. The results of this study will greatly facilitate molecular breeding of rice cultivars with ideal plant type in the future.

## Materials and Methods

### Plant materials

Minghui 63 (MH63), the male parent of the widely cultivated hybrid *indica* rice variety Shanyou63, which is distributed over a wide area spanning more than 21 longitudes and 20 latitudes in China [19], was crossed with 02428. This typical *japonica* line, with a neutral allele at the major locus *S*^*5*^, controlling hybrid sterility in most inter-subspecies crosses, as well as tolerance to low CO_2_ stress, was isolated from mutant progenies from a cross between two landraces, Pang-Xie-Gu and Ji-Bang-Dao [20]. The F_1_ hybrids of MH63 × 02428 were then consecutively selfed until the F_8_ generation to produce a set of 262 recombinant inbred lines (RILs) [21].

A germplasm panel of 1,129 accessions (S1 Table) randomly chosen from the 3K Rice Genomes Project [18] was adopted in this study to mine favorable alleles and to confirm the results of QTL mapping.

### Planting and phenotyping

All of the above plant materials were transplanted in the field at a spacing of 13.2 cm between individuals and 25 cm between rows, with a final planting density of approximately 18,000 individuals per 667 m^2^, at the Experimental Station of the Institute of Crop Sciences, Chinese Academy of Agricultural Sciences (ICS, CAAS) at Beijing (40.2°N, 116.2°E) and Sanya (18.3°N, 109.3°E) of Hainan province, as well as the Experimental Station of the Institute of Agricultural Genomics, Chinese Academy of Agricultural Sciences at Shenzhen (22.6°N, E114.5°E) of Guangdong province. The RILs, the two parents, and the germplasm panel were planted in a random complete block design with two replications.

Phenotyping of rolled leaf traits was performed as previously described [22]. The top two leaves of three main tillers per individual plant were measured for leaf width (LW) and distance between leaf boarders (LN) at the widest part of each leaf. At least five individuals per line were measured for the RILs, the two parents, and the germplasms panel. The LRI was calculated using the following formula: LRI (%) = (LW-LN)/LW × 100.

### Genotyping and mapping

Genomic DNA from MH63, 02428, and the RILs in the F_8_ generation was isolated using a DNeasy mini Kit (Qiagen), and the genotypes of the RILs were determined based on SNPs generated from whole genome sequencing with an Illumina Genome Analyzer IIx as described previously [23].

Minghui 63 (MH63) and 02428 were submitted to whole genome re-sequencing, and a total of 5,336,108,154 and 5,562,905,674 bp sequences were obtained, respectively. Alignment analysis was carried out using the MSU6.1 assembly of the Nipponbare sequence as the reference genome. A total of 5,062,106,567 bp and 5,278,080,725 bp of consistent sequences were obtained for MH63 and 02428, covering 96.57% and 94.03% of the whole genome, respectively. Single nucleotide polymorphisms (SNPs) were then identified based on these two consistent sequences to obtain an SNP dataset. A total of 48,498, 42,124, and 36,410 SNP loci were found between MH63 and 02428 with supporting evidence from more than three, four, and five reads, respectively. Since a new version of Nipponbare assembly has been available after the accomplishment of this step, we later re-mapped all the reads to the Os-Nipponbare-Reference-IRGSP-1.0 [24]. All the following works were carried out based on this new version of reference genome.

A total of 384 SNPs that are evenly distributed along the genome were used to design an Illumina SNP chip [25] for genotyping of all 262 RILs using their parents and the F_1_ populations as controls to build up a frame map. The frame map was constructed with IciMapping, version 3.3 [26]. Further mapping was carried via RAD sequencing [27] of each RIL as well as the two parents. Ultimately, a total of 58,936 qualified SNP consisting of 4,568 chromosome bins were identified and integrated into the frame map, with an average distance of 77 kb between adjacent markers.

The germplasm panel was re-sequenced with an average depth of more than 10X [18]. The reads were mapped to the reference genome of Nipponbare, and 14M high-quality SNPs were identified [18]. Based on these 14M markers, 2.9M SNPs related to potential protein-coding areas were carefully selected for further development of the 50k microarray chips. In order to build an SNP set for primary association studies in which the locations of the SNPs were independent of the SNPs chosen for microarray chip design, 27,921 SNPs were selected from the 2.9M SNPs by choosing one SNP per 100 counts.

### Data analysis

The ICIM mapping module from the V.3.3 package of QTL IciMapping [26] was used to detect the main-effect QTLs underlying the rolled leaf trait in the RILs. The default setting of LOD 2.5 was adopted as the threshold for identifying a putative locus.

Comparative mapping was carried out against a reference sequence map, GRAMENE annotation sequence map 2009 [28], to compare the QTLs detected in this study with previously reported QTLs or genes known to be associated with the rolled leaf trait in rice.

The basic scenario of compressed mixed linear model [29] implemented in the Genomic Association and Prediction Integrated Tool (GAPIT) Version 2 [30] was adopted for association analysis between QTL-flanking markers and LRI for the germplasm panel. To minimize the possible effects of population structure, the parameter of Model.selection in GAPIT was set as TRUE. Under this condition, a forward model selection by the Bayesian information criterion (BIC) was conducted to determine the optimal parameters of principal components for the LRI data. A relatively stringent threshold was adopted to identify significant correlation between the SNP and LRI with a -LOG_10_(P) value of 5.0. To minimize to the possibility of type II errors in QTL detection [31], a relatively low threshold of -LOG_10_(P) = 2.5 was also adopted with supporting evidence from linkage mapping or comparative mapping.

The allelic effects were estimated by setting the Major.allele.zero = TRUE in GAPIT Version 2 to identify the donors of favorable alleles and their effects on LRI.

## Results

### Distribution of LRI in the MH63 × 02428 RIL population and the germplasm panel

**Fig 1:**
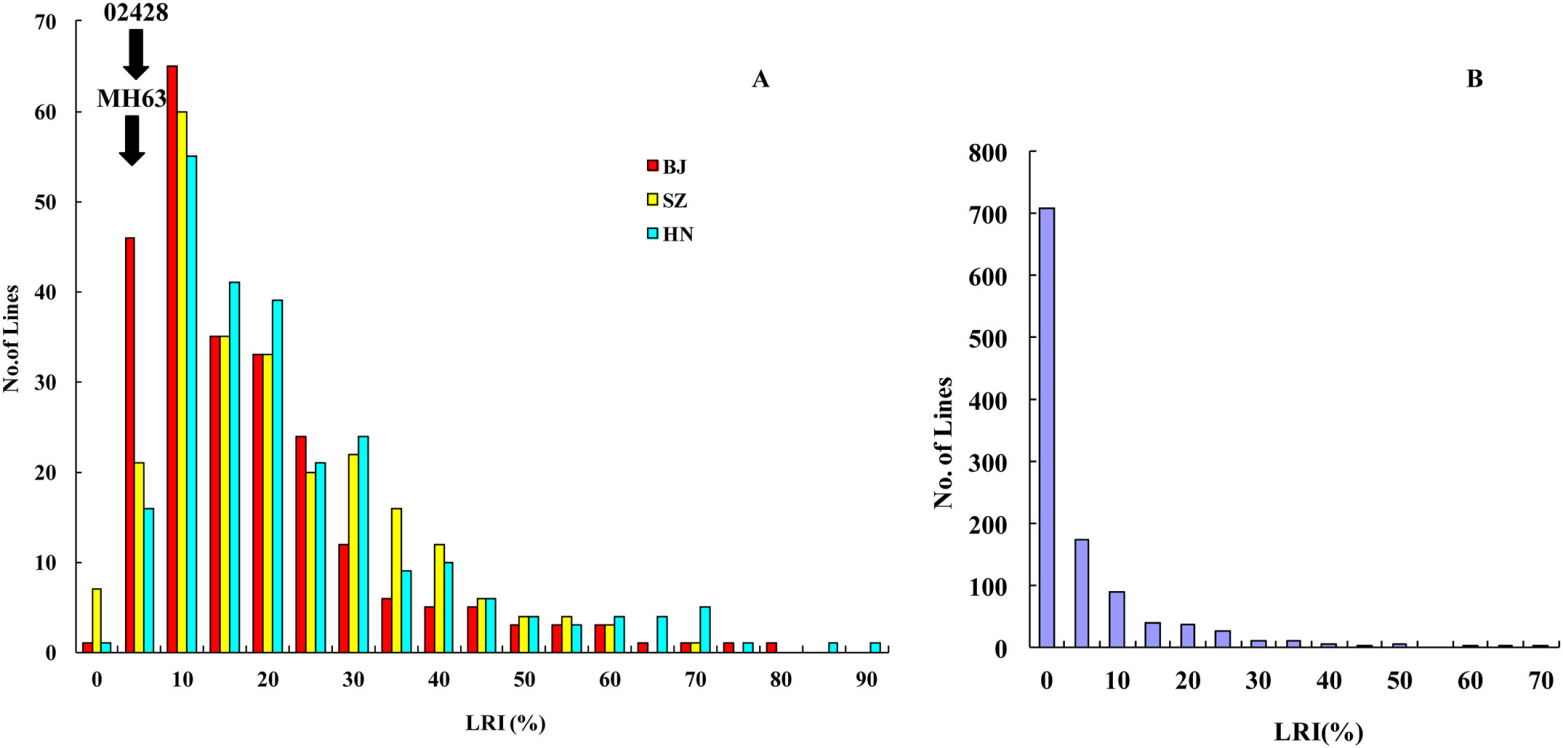
Distribution of leaf rolling index (LRI, %) in the MH63 × 02428 RIL population in three environments (A, BJ = Beijing, SZ = Shenzhen, and HN = Hainan) and a random subset of the 1127 germplasm panel at SZ (B). The average values throughout three environments of the two parents (02428 and MH63) for the RILs are indicated by two black arrows.

As shown in Fig 1A, the RIL population exhibited a similar pattern of distribution of LRI traits throughout the three environments i.e., Beijing (BJ), Shenzhen (SZ), and Hainan (HN). LRI appeared to be relatively stable in all three environments. On the other hand, even though the LRI trait did not significantly differ between the two parents (both were less than 10%), highly transgressive variations were still available in the progenies (ranging from 0–90%). However, in the germplasm panel, the variation was slightly smaller, with the LRI ranging from 0–70%, as shown in Fig 1B.

### Linkage mapping of main-effect QTLs controlling the rolled leaf trait

**Fig 2:**
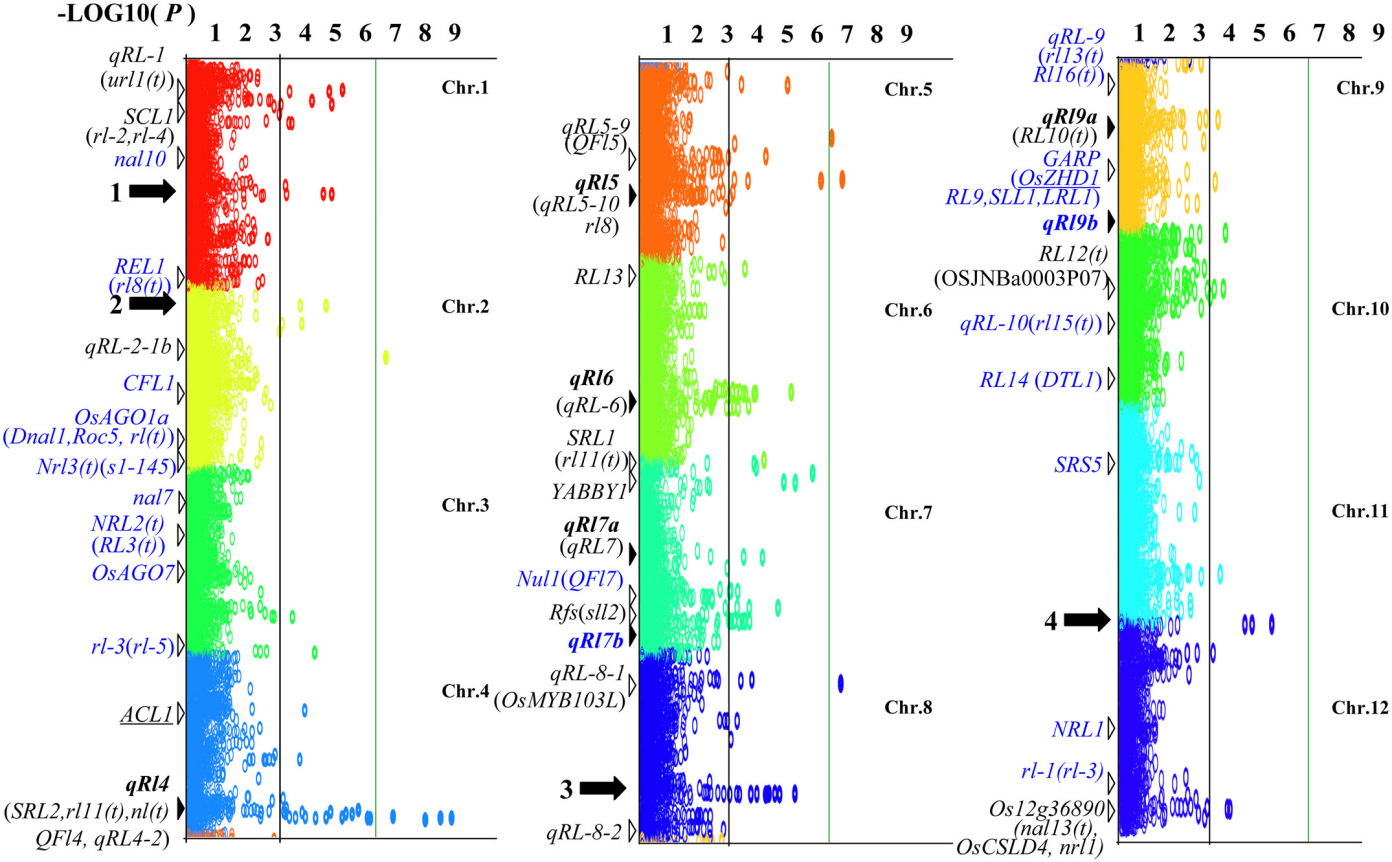
Distribution and comparison of QTLs for leaf-rolling index (LRI, %) identified by linkage mapping and GWAS (Manhattan plot) with those detected in previous studies. The reported loci/genes are indicated by white triangles and/or plain italic font, and QTLs detected by linkage mapping in our RIL population of MR63/02428 are indicated by black triangles and bold italic font. Several loci/genes with no significant support in the germplasm panel are shown in blue font. Five positions with possible new loci detected only by GWAS in the germplasm panel are indicated by black arrows and are numbered 1–5.

A total of seven main-effect QTLs (*qRl4*, *qRl5*, *qRl6*, *qRl7a*, *qRl7b*, *qRl9a*, and *qRl9b*) affecting LRI were detected on chromosomes 4, 5, 6, 7, and 9 by linkage mapping in the MH63 × 02428 RIL population across the three environments (Table 1, Fig 2). Among these, four QTLs (*qRl4*, *qRl5*, *qRl6*, and *qRl9b*) were stably expressed across all three environments, *qRl7a* and *qRl7b* were significant in only two environments (HN & SZ or SZ & BJ), and *qRl9a* was specifically expressed at Sanya of Hainan. Although the locus effects varied in different environments, the direction of gene effects on the LRI remained consistent. Among these loci, the alleles at *qRl4*, *qRl5*, *qRl7b*, and *qRl9a* from the *japonica* parent, 02428, increased the LRI, while the 02428 alleles at the three other loci (*qRl6*, *qRl7a*, and *qRl9b*) reduced the LRI in all three environments. These reverse effects of alleles from two parents at different loci may ultimately be responsible for the nearly flat leaves of MH63 and 02428.

**Table 1:** QTLs controlling leaf-rolling index (LRI) detected by linkage mapping in an RIL population at three planting sites (BJ = Beijing, HN = Hainan, and SZ = Shenzhen).

QTL	Chr	Pos (cM)	Flanking marker	HN			SZ			BJ			Reference ^3)^
				LOD	*A* (%) ^1)^	PVE (%) ^2)^	LOD	*A* (%)	PVE (%)	LOD	*A* (%)	PVE (%)	
*qRl4*	4	103	M110-Bin_1720	3.8	3.6	4.7	4.9	2.4	4.8	4.1	3.5	6.2	*rl11(t)* [51], *SRL2* [50]
*qRl5*	5	98	M129-Bin_1976	9.2	6	12.9	9.5	3.6	10.4	3.9	3.7	6.6	*qRL5-10* [22], *rl8(t)* [38]
*qRl6*	6	78	M153-Bin_2361	4.1	-3.5	6.4	5	-2.4	4.6	3.2	-3.3	3.8	*qRL-6* [32]
*qRl7a*	7	46	Bin_2619-M163	5	-4.3	6.7	3.9	-2.2	4.1				*qRL-7* [32]
*qRl7b*	7	112	M177-Bin_2847				3	3	4.5	3.7	3.6	4.5	
*qRl9a*	9	28	Bin_3289-M198	3.7	3.6	4.5							*rl13(t)* [55]
*qRl9b*	9	79	M205-Bin_3476	6.3	-4.7	8.4	3.3	-2	3.3	3.6	-3.6	6.2	

^1)^ The additive effect results from the effect of substitution of MH63 alleles with 02428 alleles.

^2)^ Phenotypic variance explained.

^3)^ Numbers in brackets are reference numbers, as listed in the reference section.

The known loci/genes for the rolled leaf trait are as follows (some genes for abaxial rolling are underlined): *qRL-1*[32], *url1(t)*[33], *rl-4*(*rl-2*)[34], *SCL1*[35], *nal10*[36], *REL1*[37], *rl8(t)* [38], *qRL-2-1b*[32], *CFL1*[39], *DNAL1*[40], *rl(t)*[41], *OsAGO1a*(*LOC_Os02g45070*)[42], *Roc5*[43], *Nrl3(t)*[44], *s1-145* [45], *nal7*(*Os03g0162000*)[46], *NRL2(t)* [47], *RL3(t)*[44], *OsAGO7*[48], *rl-3*(*rl-5*)[34], *ACL1*[49], *SRL2*[50], *rl11(t)*[51], *nl(t)*[52], *qRL4-2*[22], *QFl4* and *QFl5*[53], *qRL5-9* and *qRL5-10*[22], *rl8* [54], *RL13*[55], *qRL-6*[32], *sd-sl*[56], *rl11(t)*[51], *SRL1*[57], *YABBY1*[8], *rfs*[34], *qRL-7*[32], *Nul1*[58], *QFl7*[53], *sll2*[59], *qRL-8-1*, *qRL-8-2* and *qRL-9*[32], *OsMYB103L*[60], *rl13(t)*[55], *Rl16(t)*[61], *RL10(t)*[62], *SLL1*[63], *RL9*(*rl9(t)*,*GARP*)[64], *OSZHD1* [65], *LRL1*[66], *QFl9*[53], *OSJNBa0003P07*[67], *RL12(t)*[68], *rl15(t)*[69], *qRL-10*[32], *DTL1*[70], *RL14*[71], *SRS5*[72], *NRL1*(*OSJNBa0027H05*)[73], *rl-1*(*rl-3*)[34], *nal3(t)*[74], and *nrl1*[46].

### GWAS mapping of QTLs affecting the rolled leaf trait

A total of 18 significant loci were detected by GWAS using a combination of the relatively stringent threshold of -LOG_10_(P) = 5.0 and the relatively low threshold of -LOG_10_(P) = 2.5, with supporting evidence from either our linkage mapping with RILs or previous reports (Table 2, Fig 2). These QTLs are distributed throughout the genome, except for chromosome 3, 10, and 11. Of these QTLs, 14 (77.8%) are closely related to loci that were previously identified by comparative mapping or to QTLs identified by our linkage mapping of the MH63 × 02428 RIL population, whereas the other four (*gRl1-2*, *gRl5-1*, *gRl5-2*, and *gRl12-1*) are newly identified QTLs that are associated with LRI.

**Table 2:** QTLs affecting leaf-rolling index (LRI) detected by GWAS in a panel of 1,129 germplasms.

QTL	Range (bp)	-LOG10(*P*)	QTL from linkage mapping	QTL from references
*gRl1-1*	4,832,637–4,832,637	5.3		*qRL-1* [32], *url1(t)* [33], *rl-4(rl-2)* [34], *SCL1* [35]
*gRl1-2*	25,404,548–25,404,548	5.7		
*gRl2-1*	7,519,002–9,022,802	3.9		*qRL-2-1b* [40]
*gRl2-2*	14,150,759–20,850,818	6.8		*CFL1* [41]
*gRl4-1*	19,823,372–20,860,914	3.8		*ACL1* [49]
*gRl4-2*	31,977,228–32,592,463	9.1	*qRl4*	*rl11(t)* [51], *SRL2* [50], *qRL4-2* [22], *nl(t)* [52]
*gRl5-1*	3,117,099–3,117,099	5		
*gRl5-2*	11,228,990–11,228,990	6.6		
*gRl5-3*	17,598,480–19,569,643	6.9		*qRL5-9* [22]
*gRl5-4*	20,444,240–21,926,430	3.1	*qRl5*	*qRL5-10* [22], *rl8(t)* [38]
*gRl6-1*	20,532,308–22,944,285	5.1	*qRl6*	*qRL-6* [32]
*gRl7-1*	1,762,263–3,230,532	5.9		*YABBY1* [8]
*gRl7-2*	14,684,286–16,298,897	4.2	*qRl7a*	*qRL-7* [32]
*gRl7-3*	27,671,916–27,671,916	2.6	*qRl7b*	
*gRl8-1*	4,426,494–4,426,494	6.9		*qRL-8-1* [32]
*gRl8-2*	26,810,755–27,101,483	5.3		*qRL-8-2* [32]
*gRl9-1*	7,959,893–10,895,719	3.3	*qRl9a*	*rl13(t)* [55]
*gRl12-1*	363,480–363,480	5.2		

### Allele mining for the rolled leaf trait

We mined favorable alleles and estimated their effects on LRI using a random subset of the 1127 3K panel. We ultimately detected a total of 33 favorable alleles for the 14 loci, which were consistently detected in GWAS and linkage mapping or comparative mapping (Table 3). Among these, 16 (48.5%) alleles were found in the five regions (*gRl4-2*, *gRl5-4*, *gRl6-1*, *gRl7-2*, and *gRl7-3*) associated with loci identified by linkage mapping of the RIL population, and 24 (72.7%) of the 33 alleles were donated by the favorable germplasms from the 3K Rice Genomes Project panel (Table 3). The average effect of the favorable alleles on LRI was 1.9%, ranging from 1.1% to 3.1%. Approximately 30.3% of the favorable alleles can improve the LRI by no less than 2%, with the maximum effects from the A alleles at the representative SNP at the position of 14,150,759 in the region of *gRl2-2*.

**Table 3:** Representative SNPs for favorable alleles and their effects on leaf rolling index (LRI, %) simultaneously detected by GWAS and linkage mapping in this study or previous studies.

QTL	QTL from linkage mapping	Physical position (bp)	Favorable SNP allele	Effect (%)	Top five accessions with favorable alleles ^1)^
*gRl1-1*		4,832,637	T	2.1	IRIS_313–9759 (65.2), IRIS_313–8023 (60.3), IRIS_313–8027 (57.3), IRIS_313–8149 (55.4), IRIS_313–8129 (49.9)
*gRl2-1*		7,519,002	G	1.7	IRIS_313–9759 (65.2), IRIS_313–8023 (60.3), IRIS_313–8027 (57.3), IRIS_313–8075 (55.6), IRIS_313–8149 (55.4)
		9,022,802	T	1.6	IRIS_313–9759 (65.2), IRIS_313–8023 (60.3), IRIS_313–8027 (57.3), IRIS_313–8075 (55.6), IRIS_313–8149 (55.4)
*gRl2-2*		14,150,759	A	3.1	IRIS_313–9759 (65.2), IRIS_313–8023 (60.3), IRIS_313–8027 (57.3), IRIS_313–8075 (55.6), IRIS_313–8149 (55.4)
		20,850,818	A	1.5	Nipponbare
*gRl4-1*		19,823,372	T	1.6	IRIS_313–9759 (65.2), IRIS_313–8023 (60.3), IRIS_313–8027 (57.3), IRIS_313–8075 (55.6), IRIS_313–8149 (55.4)
		20,743,867	A	1.9	IRIS_313–9759 (65.2), IRIS_313–8023 (60.3), IRIS_313–8027 (57.3),IRIS_313–8149 (55.4), IRIS_313–8129 (49.9)
		20,786,774	T	1.4	IRIS_313–9759 (65.2), IRIS_313–8023 (60.3), IRIS_313–8027 (57.3),IRIS_313–8149 (55.4), IRIS_313–8129 (49.9)
		20,821,210	C	1.3	IRIS_313–9759 (65.2), IRIS_313–8023 (60.3), IRIS_313–8027 (57.3),IRIS_313–8149 (55.4), IRIS_313–8129 (49.9)
		20,860,914	A	1.3	IRIS_313–9759 (65.2), IRIS_313–8023 (60.3), IRIS_313–8027 (57.3),IRIS_313–8149 (55.4), IRIS_313–8129 (49.9)
*gRl4-2*	*qRl4*	31,977,228	A	2.4	Nipponbare
		32,086,234	A	1.1	IRIS_313-8135(47.0), B153 (28.1), CX15 (46.5), CX28 (35.0), CX314 (42.0)
		32,134,331	A	1.8	IRIS_313–8023 (60.3), IRIS_313–8027 (57.3), IRIS_313–8075 (55.6), IRIS_313–8149 (55.4), IRIS_313–8111 (48.7)
		32,227,059	G	2.4	IRIS_313–9759 (65.2), IRIS_313–8023 (60.3), IRIS_313–8027 (57.3), IRIS_313–8075 (55.6), IRIS_313–8149 (55.4)
		32,236,238	A	1.7	IRIS_313–9759 (65.2), IRIS_313–8023 (60.3), IRIS_313–8027 (57.3), IRIS_313–8075 (55.6), IRIS_313–8149 (55.4)
		32,271,965	C	2.7	IRIS_313–9759 (65.2), IRIS_313–8023 (60.3), IRIS_313–8027 (57.3), IRIS_313–8075 (55.6), IRIS_313–8149 (55.4)
		32,592,463	C	2.5	IRIS_313–9759 (65.2), IRIS_313–8023 (60.3), IRIS_313–8027 (57.3), IRIS_313–8075 (55.6), IRIS_313–8149 (55.4)
*gRl5-3*		17,598,480	T	2.4	IRIS_313–9759 (65.2), IRIS_313–8023 (60.3), IRIS_313–8027 (57.3), IRIS_313–8075 (55.6), IRIS_313–8149 (55.4)
*gRl5-4*		19,471,779	T	2.1	Nipponbare
	*qRl5*	19,569,643	C	2.1	Nipponbare
*gRl6-1*	*qRl6*	20,532,308	G	2.2	IRIS_313–8023 (60.3), IRIS_313–8027 (57.3), IRIS_313–8149 (55.4), IRIS_313–8129 (49.9), IRIS_313–8185 (49.8)
		22,661,800	G	1.8	IRIS_313–9759 (65.2), IRIS_313–8023 (60.3), IRIS_313–8075 (55.6), IRIS_313–8149 (55.4), IRIS_313–8129 (49.9)
		22,854,876	T	1.4	IRIS_313–9759 (65.2), IRIS_313–8149 (55.4), IRIS_313–8129 (49.9), IRIS_313–8075 (55.6), IRIS_313–8023 (60.3)
		22,944,285	T	2.6	IRIS_313–10084 (36.9), CX15 (46.5), CX288 (26.5), CX314 (42.0), CX361 (14.6)
*gRl7-1*		1,762,263	C	2.3	IRIS_313–9759 (65.2), IRIS_313–8023 (60.3), IRIS_313–8027 (57.3), IRIS_313–8075 (55.6), IRIS_313–8149 (55.4)
		3,230,532	T	1.9	Nipponbare
*gRl7-2*	*qRl7a*	14,684,286	C	1.7	Nipponbare
		16,298,897	A	1.4	IRIS_313–8023 (60.3), IRIS_313–8075 (55.6), IRIS_313–8185 (49.8), IRIS_313–8111 (48.7), B055 (48.0)
*gRl7-3*	*qRl7b*	27,671,916	T	1.4	Nipponbare
*gRl8-1*		4,426,494	C	2.9	IRIS_313–9759 (65.2), IRIS_313–8075 (55.6), IRIS_313–8149 (55.4), IRIS_313–8185 (49.8), IRIS_313–10083 (37.2)
*gRl8-2*		21,374,656	G	2.3	Nipponbare
*gRl9-1*		7,959,893	C	1.9	Nipponbare
		10,895,719	G	1.3	IRIS_313–9759 (65.2), IRIS_313–8075 (55.6), IRIS_313–8149 (55.4), IRIS_313–8129 (49.9), IRIS_313–8111 (48.7)

^1)^ The accession ID can be found in the database (http://www.rmbreeding.cn/snp3k) for further details. Numbers in brackets are LRI values (%).

### Subregional analysis of *gRl4-2*

The region of *gRl4-2* possessed the highest peak among all the rolled leaf loci detected by GWASin this study. The *gRl4-2* was also consistently detected in the RILs derived from MH63 × 02428 across three environments. Whether this clustering was caused by tightly linked loci or a single locus remains unclear. To get more details in the candidate region. we extracted 89,349 SNP markers surrounding the above peaks with an average distance of 18.1 ± 55.2 bp between adjacent markers to perform a fine re-scanning of this region and to re-estimate all of the -LOG10(P) values and additive effects.

**Fig 3:**
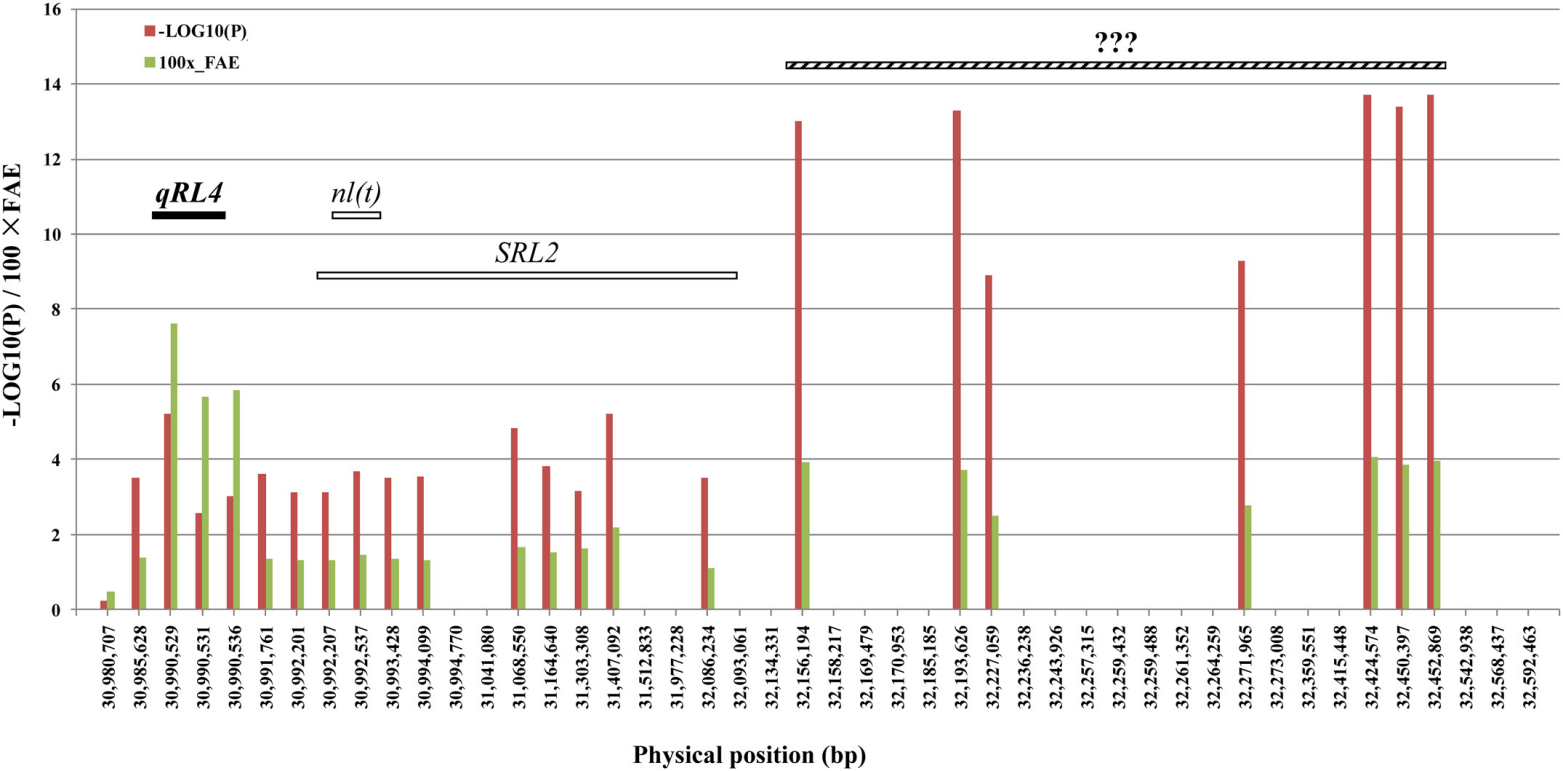
Subregional analysis of the region harboring *gRl4-2* by increasing marker density. The known loci, including the QTLs detected by linkage mapping in the MH63 × 02428 RIL population, the loci from the literature, and (likely) new clusters within subregions gRl4-2_1, gRl4-2_2, and gRl4-2_3 are indicated by horizontal black, white, and striped bars, respectively. The vertical bars in red and green indicate the—LOG10(P) and 100× favorable allele effect (FAE) values of the peak markers, respectively.

It’s notable that within this region of no more than 2 Mb (30,977,335–32,592,463 bp) at the end of the long arm of chromosome 4, at least three clusters of peaks were found (Fig 3). The first subregion (*gRl4-2*_1) covers nucleotides in a range from 30,980,707 to 30,994,770 bp (marked by an SNP peak of -LOG10(P) = 5.2), which is consistent with our linkage mapping-derived locus *qLR4*, with an average value of 2.9% (ranging from 1.3–7.6%) favorable effects on LRI. The second subregion (*gRl4-2*_2) is marked by an SNP peak of -LOG10(P) = 5.2 and comprises nucleotides in the range of 31,068,550–32,086,234 bp. The favorable allele effects averaged 1.6% (ranging from 1.1–2.2%) for LRI. The region *gRl4-2*_2 harbors two previously reported genes, *nl(t)*[52] and *SRL2*[50]. The third subregion (*gRl4-2*_3) is located at the physical range of 32,156,194–32,452,869 bp, with an SNP peak of -LOG10(P) = 13.7. The favorable allele effects averaged 3.5%, ranging from 2.4–4.1% for LRI, suggesting that a new locus or loci could be localized within this region.

## Discussion

### QTLs underlying segregation of the rolled leaf trait in the RIL population

In this study, we carried out both linkage mapping and GWAS analysis in order to perform accurate locus searching and to mine multiple favorable alleles for the rolled leaf trait, one of the key components of plant architecture. The rolled leaf trait showed transgressive segregation in RILs derived from two parents with insignificant differences in this trait (LRI of no more than 10%, Fig 1). As shown in Table 1, favorable alleles at the detected QTLs are evenly dispersed in the two parents. Therefore, transgressive segregation of the rolled leaf trait in the RIL population can be partially explained by the reverse patterns of the allelic effects at the seven loci. Our results further support the observation that even though the germplasms themselves do not show prominent traits, they do harbor some excellent alleles, as previously observed for grain yield [75], salt tolerance [76], cold tolerance [13], and drought tolerance [12].

Of the seven loci identified for the rolled leaf trait, five were previously reported, and according to the comparative mapping, four of the above loci (*qRl4*, *qRl5*, *qRl6*, and *qRl7a*) are close to the previously reported loci *rl1(t)* [51], *qRL5-10* [22], *qRL-6* [32], and *qRL-7* [32], respectively. Two QTLs (*qRl7b* and *qRl9b*) are newly reported; the former was confirmed by GWAS analysis.

Previous studies have revealed multiple locus clusters, including regions on chromosome 4, 9, and 12 (Fig 2). Here, we found clustering of allele peaks based on the GWAS results, especially in *gRl4-2* at the end of chromosome 4, where at least three clusters of allele peaks were detected in the random subset of the 3K Rice germplasms panel (Fig 3).

### Comparison of rolled leaf QTLs detected by GWAS with those revealed in earlier studies

Approximately six (33.3%) of the 18 GWAS loci were confirmed by our linkage mapping with an RIL population, five of which were previously reported (Table 2). An additional eight loci were supported by comparative mapping with the results from earlier reports. Taking together, these 14 (77.8%) GWAS loci are fairly reliable and appropriate for use in allele mining for breeding purposes. We identified at least 33 key SNP genotypes for favorable alleles at these loci, with an average of 2.4 SNP genotypes at each locus (Table 3).

Specifically, *gRl1-1* on chromosome 1 is located in the same region as the previously reported rolled leaf loci *qRL-1*, *url1(t)*, *rl-4*(*rl-2*), and *SCL1* [32–35]. Two loci (*gRl2-1* and *gRl2-2*) on chromosome 2 were mapped together with *qRL-2-1b* [32] and *CFL1* [39], respectively. We identified two loci (*gRl4-1* and *gRl4-2*) on chromosome 4: the former is located in the same region as *ACL1*, for abaxial rolled leaves [49], and the latter is located in a region harboring *SRL2*[50], *rl11(t)* [51], *nl(t)*[52], and *qRL4-2* [22]. The latter locus also harbors QTL *qRl4*, as detected in our linkage map (Table 1). Two loci (*gRl5-3*, and *gRl5-4*) mapped together with *qRL5-9* [22], *qRL5-10* [22], and *rl8* [54], respectively, and *gRl5-4* was also detected on our linkage map (Table 1). Only one locus (*gRl6-1*) on chromosome 6, which was mapped together with *qRl6* detected by linkage mapping, was mapped in the same region with *qRL-6* from a previous report [32]. There are two significant loci on chromosome 7 and 8, respectively. Among these, *gRl7-1* and *gRl7-2* were mapped together with the loci *YABBY1* [8] and *qRL-7* [32] from previous reports, and *gRl8-1* and *gRl8-2* were mapped to the same regions as previously reported loci *qRL-8-1* and *qRL-8-2* [32], respectively. Only one locus (*gRl9*) on chromosomes 9 was mapped together with *rl13(t)* from a previous study [55]. All of these candidate loci affecting the rolled leaf trait detected in different experiments are reliable and suitable for use in molecular breeding for plant type. Four loci (*gRl1-2*, *gRl5-1*, *gRl5-2*, and *gRl12-1*) were identified for the first time in this study and likely represent new QTLs for the rolled leaf trait.

To further explore the SNP markers with extremely higher density (average 18.1 ± 55.2 bp between adjacent markers), we split *gRl4-2* on chromosome 4 into at least three subregions. The second region, which is located in the region 31,068,550–32,086,234, was previously reported as *nl(t)* and *SRL2* [50,52]. The natural variations affect the LRI at a level of 1.7–2.2%, without significant correlation to leaf width, as detected in our diverse germplasm panel, although mutant alleles, especially *nl(t)* at this locus, cause narrow leaves in addition to rolled leaves. The third region, located at 32,156,194–32,452,869, is thought to harbor new QTLs underlying the rolled leaf trait, with gene effects as high as 3.5% of the average gene effects for LRI.

By analyzing a highly diverse germplasm panel, we detected many new alleles, such as multiple alleles at the *ACL1* locus [49], in which the *ACL1* mutant has abaxial leaf rolling, while at least five favorable alleles from our 3K Rice germplasms improve the LRI by an average of 1.5%.

### Implications for rice breeding for ideal plant type

The rolled leaf trait is a morphological character for which tremendous genetic variation exists among different rice genotypes, as shown in the current study. Suitably rolled leaves may allow rice plants to have greater effective leaf area per unit land without causing shading, thus likely resulting in extremely high yields due to higher rates of photosynthesis, as demonstrated in super hybrid rice [1]. Cultivars with moderately rolled leaves are suitable for relatively high density cultivation [11] and thought to be with better lodging and disease resistances at the population level [10]. Moreover, genotypes with partially rolled leaves may have better water use efficiency because rolled leaves are expected to have reduced leaf area [77]. Although both MH63 and 02428 have nearly flat leaves, the derived RILs showed various degrees of leaf rolling due to recombination of non-allelic parental alleles. Indeed, the most favorable LRI for *indica* cultivars is approximately 12% [78]. Therefore, it is possible to improve the leaf type of existing elite varieties by identifying “hidden” favorable alleles segregating in existing breeding populations and germplasms and introgressing and pyramiding them into elite backgrounds by MAS. The favorable alleles from IRIS_313–8023, IRIS_313–8027, IRIS_313–8149, IRIS_313–8129, and IRIS_313–8185, and perhaps even Nipponbare, at five QTLs (*qRl4*, *qRl5*, *qRl6*, *qRl7a*, and *qRl7b*) for the rolled leaf trait consistently identified in the RILs and in the re-sequenced germplasms (Table 3) in this study and in previous studies could be used to deploy allele combinations for ideal plant type in rice by MAS.

## Conclusion

We identified seven main-effect QTLs underlying the transgressive segregation of the rolled leaf trait in rice in progenies from parents with minor differences in this trait. Five of these QTLs were previously reported, two (*qRl7b* and *qRl9b*) are newly identified, and one, qRl7b, was confirmed by GWAS analysis. Eighteen loci were found by GWAS: four are newly identified and the 14 other loci are consistent with QTLs from linkage mapping or comparative mapping. We carried out favorable allele mining for these 14 loci and identified possible elite donors for future plant type breeding programs. By performing subregional analysis, we identified a subregion (*gRl4-2_3*) with a possible new locus/loci and favorable alleles with an average effect of 3.5% for LRI, ranging from 2.4 to 4.1%. The favorable alleles at five QTLs (*qRl4*, *qRl5*, *qRl6*, *qRl7a*, and *qRl7b*) for the rolled leaf trait that were consistently identified in different populations could be used for breeding rice with ideal plant type by MAS.

## Supporting Information

S1 Table List of accessions used in this study. (DOC)
