# Supplementary material for: Joint Mapping and Allele Mining of the Rolled Leaf Trait in Rice (Oryza sativa L.)
Source: PLoS One. 2016 Jul 21;11(7):e0158246. doi: 10.1371/journal.pone.0158246 (PMC4956317; doi:10.1371/journal.pone.0158246)
Supplement: S1 Table — (DOC) [file pone.0158246.s001.doc]

**S1Table List of accessions used in this study**

| **Acc_ID ^1)^** | **LRI (%)** | **Type ^2)^** | **Origin** |
| --- | --- | --- | --- |
| IRIS_313-8113 | 0.0 | temp | N.A. |
| IRIS_313-8065 | 0.0 | temp | N.A. |
| IRIS_313-9996 | 19.5 | trop | South Korea |
| IRIS_313-10353 | 18.2 | ind | South Korea |
| IRIS_313-8665 | 11.9 | temp | United States |
| IRIS_313-10242 | 1.5 | temp | Hungary |
| IRIS_313-11422 | 0.7 | ind | China |
| IRIS_313-11585 | 1.0 | temp | China |
| IRIS_313-12273 | 2.5 | ind | China |
| IRIS_313-10854 | 4.3 | aus | India |
| IRIS_313-11290 | 0.0 | adm | India |
| IRIS_313-10849 | 4.5 | aus | India |
| IRIS_313-11056 | 2.4 | aus | Bangladesh |
| IRIS_313-11058 | 0.0 | aus | Bangladesh |
| IRIS_313-11059 | 2.6 | aus | Bangladesh |
| IRIS_313-11348 | 0.6 | aus | India |
| IRIS_313-11596 | 0.5 | ind | India |
| IRIS_313-11014 | 0.0 | aus | Bangladesh |
| IRIS_313-11702 | 0.6 | ind | United States |
| IRIS_313-11664 | 3.3 | ind | China |
| IRIS_313-10534 | 1.7 | aus | India |
| IRIS_313-11795 | 6.4 | ind | China |
| IRIS_313-11746 | 0.0 | ind | China |
| IRIS_313-11747 | 2.5 | temp | China |
| IRIS_313-11798 | 0.0 | ind | China |
| IRIS_313-11799 | 0.0 | ind | China |
| IRIS_313-11801 | 0.0 | ind | China |
| IRIS_313-11570 | 4.0 | temp | China |
| IRIS_313-11598 | 0.0 | ind | India |
| IRIS_313-11712 | 0.0 | ind | India |
| IRIS_313-11980 | 0.6 | ind | Taiwan |
| IRIS_313-11887 | 0.0 | ind | Philippines |
| IRIS_313-11385 | 0.0 | ind | N.A. |
| IRIS_313-11538 | 0.0 | trop | Philippines |
| IRIS_313-11866 | 1.0 | ind | China |
| IRIS_313-11506 | 0.0 | ind | India |
| IRIS_313-11477 | 0.0 | ind | India |
| IRIS_313-11937 | 0.0 | ind | India |
| IRIS_313-11986 | 1.1 | ind | China |
| IRIS_313-11666 | 1.0 | ind | China |
| IRIS_313-11667 | 1.6 | ind | China |
| IRIS_313-11752 | 5.9 | ind | China |
| IRIS_313-11602 | 0.0 | aus | India |
| IRIS_313-11729 | 0.0 | ind | China |
| IRIS_313-11433 | 0.0 | trop | India |
| IRIS_313-11029 | 0.0 | aus | Pakistan |
| IRIS_313-11158 | 0.6 | ind | China |
| IRIS_313-11636 | 0.0 | ind | India |
| IRIS_313-11639 | 0.0 | ind | India |
| IRIS_313-12135 | 1.3 | ind | Malaysia |
| IRIS_313-11763 | 10.6 | ind | Cameroon |
| IRIS_313-11732 | 3.5 | ind | China |
| IRIS_313-11807 | 0.0 | ind | Colombia |
| IRIS_313-11243 | 23.6 | aus | India |
| IRIS_313-11668 | 0.0 | ind | China |
| IRIS_313-11034 | 0.6 | aus | Pakistan |
| IRIS_313-11371 | 0.0 | aus | India |
| IRIS_313-11733 | 1.8 | ind | China |
| IRIS_313-11734 | 5.5 | ind | China |
| IRIS_313-11604 | 0.0 | ind | India |
| IRIS_313-11176 | 0.0 | ind | India |
| IRIS_313-12232 | 5.5 | ind | China |
| IRIS_313-10603 | 0.0 | aus | Bangladesh |
| IRIS_313-11968 | 1.0 | ind | China |
| IRIS_313-11038 | 4.9 | ind | China |
| IRIS_313-10932 | 2.6 | ind | China |
| IRIS_313-11025 | 0.0 | aus | Pakistan |
| IRIS_313-11015 | 0.0 | aus | Bangladesh |
| IRIS_313-10754 | 0.0 | ind | N.A. |
| IRIS_313-11039 | 2.1 | ind | China |
| IRIS_313-11076 | 0.0 | ind | Laos |
| IRIS_313-10736 | 0.0 | aus | Nepal |
| IRIS_313-11245 | 0.0 | ind | N.A. |
| IRIS_313-10753 | 0.0 | ind | N.A. |
| IRIS_313-11875 | 0.0 | temp | Japan |
| IRIS_313-11455 | 0.0 | aus | India |
| IRIS_313-11689 | 0.0 | adm | South Korea |
| IRIS_313-11965 | 0.0 | ind | China |
| IRIS_313-11722 | 0.0 | ind | Bangladesh |
| IRIS_313-11476 | 0.0 | aus | India |
| IRIS_313-10729 | 1.8 | adm | Ivory Coast |
| IRIS_313-11653 | 2.0 | temp | China |
| IRIS_313-10568 | 0.0 | temp | N.A. |
| IRIS_313-11804 | 0.0 | ind | China |
| IRIS_313-10642 | 1.7 | temp | N.A. |
| IRIS_313-15910 | 9.7 | adm | N.A. |
| IRIS_313-15907 | 8.8 | trop | N.A. |
| IRIS_313-15900 | 0.0 | ind | N.A. |
| IRIS_313-15899 | 2.6 | ind | N.A. |
| IRIS_313-15906 | 0.7 | ind | N.A. |
| IRIS_313-15901 | 0.0 | ind | N.A. |
| IRIS_313-15902 | 0.0 | ind | N.A. |
| IRIS_313-15904 | 3.3 | temp | N.A. |
| IRIS_313-15910 | 0.0 | adm | N.A. |
| IRIS_313-8265 | 4.8 | ind | India |
| IRIS_313-8963 | 0.0 | aus | Bangladesh |
| IRIS_313-9422 | 0.0 | aus | Bangladesh |
| IRIS_313-10352 | 0.0 | ind | Colombia |
| IRIS_313-8721 | 0.0 | aus | Bangladesh |
| IRIS_313-10403 | 0.0 | ind | Colombia |
| IRIS_313-8398 | 0.0 | aus | Pakistan |
| IRIS_313-8771 | 2.3 | aus | India |
| IRIS_313-9814 | 0.0 | adm | Hungary |
| IRIS_313-8204 | 7.1 | temp | N.A. |
| IRIS_313-8135 | 47.0 | trop | N.A. |
| IRIS_313-8064 | 0.0 | jap | N.A. |
| IRIS_313-8061 | 0.0 | jap | N.A. |
| IRIS_313-8057 | 19.7 | jap | N.A. |
| IRIS_313-8173 | 45.7 | temp | N.A. |
| IRIS_313-8209 | 8.7 | temp | N.A. |
| IRIS_313-8171 | 0.0 | temp | N.A. |
| IRIS_313-8109 | 17.1 | trop | N.A. |
| IRIS_313-8165 | 21.4 | temp | N.A. |
| IRIS_313-8085 | 11.5 | temp | N.A. |
| IRIS_313-8161 | 0.0 | temp | N.A. |
| IRIS_313-8160 | 21.4 | jap | N.A. |
| IRIS_313-8172 | 5.8 | ind | N.A. |
| IRIS_313-8099 | 19.4 | temp | N.A. |
| IRIS_313-8184 | 11.0 | ind | N.A. |
| IRIS_313-8026 | 3.5 | temp | N.A. |
| IRIS_313-8212 | 0.0 | ind | N.A. |
| IRIS_313-8185 | 49.8 | ind | N.A. |
| IRIS_313-8075 | 55.6 | trop | N.A. |
| IRIS_313-7684 | 0.0 | ind | N.A. |
| IRIS_313-7691 | 0.0 | ind | N.A. |
| IRIS_313-8186 | 0.0 | temp | N.A. |
| IRIS_313-8119 | 33.5 | temp | N.A. |
| IRIS_313-8155 | 36.6 | trop | N.A. |
| IRIS_313-8192 | 4.1 | temp | N.A. |
| IRIS_313-8033 | 22.7 | temp | N.A. |
| IRIS_313-8039 | 7.3 | temp | N.A. |
| IRIS_313-8208 | 23.0 | temp | N.A. |
| IRIS_313-8195 | 18.8 | temp | N.A. |
| IRIS_313-8090 | 27.5 | temp | N.A. |
| IRIS_313-8067 | 0.0 | temp | N.A. |
| IRIS_313-8214 | 16.0 | temp | N.A. |
| IRIS_313-8215 | 10.7 | aro | N.A. |
| IRIS_313-8050 | 13.5 | jap | N.A. |
| IRIS_313-8200 | 10.3 | temp | N.A. |
| IRIS_313-8032 | 16.1 | temp | N.A. |
| IRIS_313-8126 | 22.7 | temp | N.A. |
| IRIS_313-8127 | 27.1 | temp | N.A. |
| IRIS_313-8202 | 0.0 | temp | N.A. |
| IRIS_313-8048 | 27.3 | temp | N.A. |
| IRIS_313-8162 | 3.1 | ind | N.A. |
| IRIS_313-8053 | 9.0 | temp | N.A. |
| IRIS_313-8031 | 28.5 | trop | N.A. |
| IRIS_313-7766 | 0.0 | ind | N.A. |
| IRIS_313-8218 | 0.0 | aus | N.A. |
| IRIS_313-8052 | 26.9 | trop | N.A. |
| IRIS_313-8096 | 30.3 | temp | N.A. |
| IRIS_313-8105 | 26.9 | temp | N.A. |
| IRIS_313-8148 | 0.0 | temp | N.A. |
| IRIS_313-8097 | 7.5 | temp | N.A. |
| IRIS_313-7807 | 0.0 | ind | N.A. |
| IRIS_313-7808 | 0.0 | ind | N.A. |
| IRIS_313-7815 | 0.0 | ind | N.A. |
| IRIS_313-7816 | 0.0 | ind | N.A. |
| IRIS_313-8164 | 39.7 | jap | N.A. |
| IRIS_313-8669 | 20.2 | temp | United States |
| IRIS_313-8405 | 5.7 | ind | China |
| IRIS_313-8502 | 12.1 | trop | United States |
| IRIS_313-8435 | 0.0 | ind | India |
| IRIS_313-10083 | 37.2 | temp | Spain |
| IRIS_313-10148 | 0.0 | ind | India |
| IRIS_313-10092 | 2.1 | temp | South Korea |
| IRIS_313-9917 | 0.0 | ind | Sri Lanka |
| IRIS_313-10093 | 0.0 | temp | South Korea |
| IRIS_313-10059 | 0.0 | temp | South Korea |
| IRIS_313-9482 | 0.0 | ind | China |
| IRIS_313-8914 | 0.0 | ind | China |
| IRIS_313-10047 | 2.6 | ind | Nigeria |
| IRIS_313-8859 | 0.0 | adm | China |
| IRIS_313-8940 | 0.0 | ind | China |
| IRIS_313-10178 | 3.8 | ind | China |
| IRIS_313-10056 | 0.0 | temp | Japan |
| IRIS_313-9228 | 0.0 | temp | Japan |
| IRIS_313-10167 | 0.0 | ind | Philippines |
| IRIS_313-9922 | 0.0 | ind | South Korea |
| IRIS_313-10168 | 0.0 | ind | China |
| IRIS_313-9925 | 18.6 | ind | South Korea |
| IRIS_313-10040 | 0.0 | ind | South Korea |
| IRIS_313-10191 | 4.3 | ind | China |
| IRIS_313-10170 | 3.7 | ind | China |
| IRIS_313-10111 | 31.3 | temp | Italy |
| IRIS_313-9648 | 0.0 | adm | United States |
| IRIS_313-9570 | 6.1 | ind | China |
| IRIS_313-9065 | 0.0 | ind | China |
| IRIS_313-8873 | 0.0 | temp | Taiwan |
| IRIS_313-8856 | 20.0 | temp | United States |
| IRIS_313-10084 | 36.9 | temp | Spain |
| IRIS_313-10000 | 18.6 | ind | South Korea |
| IRIS_313-9964 | 0.0 | temp | Georgia |
| IRIS_313-9961 | 0.0 | temp | Norway |
| IRIS_313-9111 | 0.0 | ind | China |
| IRIS_313-9891 | 3.4 | temp | South Korea |
| IRIS_313-9253 | 0.0 | ind | China |
| IRIS_313-10247 | 0.0 | ind | Cuba |
| IRIS_313-10289 | 2.5 | ind | Venezuela |
| IRIS_313-10333 | 5.7 | ind | Indonesia |
| IRIS_313-10293 | 0.0 | jap | Cuba |
| IRIS_313-10274 | 0.0 | ind | Guatemala |
| IRIS_313-10314 | 6.7 | trop | Ecuador |
| IRIS_313-10366 | 0.0 | ind | Philippines |
| IRIS_313-10398 | 0.0 | ind | Colombia |
| IRIS_313-8399 | 42.9 | temp | France |
| IRIS_313-8433 | 0.0 | ind | China |
| IRIS_313-8444 | 17.8 | temp | United States |
| B166 | 9.6 | temp | Korea |
| B167 | 0.0 | temp | Korea |
| B001 | 25.8 | temp | N.A. |
| B002 | 0.0 | temp | N.A. |
| B003 | 0.0 | temp | N.A. |
| B004 | 0.0 | temp | N.A. |
| B005 | 0.0 | temp | N.A. |
| B169 | 0.8 | temp | Japan |
| B009 | 3.9 | ind | Vietnam |
| B010 | 0.0 | ind | Malaysia |
| B013 | 0.0 | ind | N.A. |
| B170 | 8.8 | temp | Soviet Union |
| B016 | 0.0 | temp | N.A. |
| B028 | 0.0 | ind | N.A. |
| B029 | 0.0 | ind | N.A. |
| B035 | 37.9 | ind | Usa |
| B039 | 6.8 | adm | C脭te Divoire |
| B040 | 0.0 | ind | Uganda |
| B181 | 0.0 | ind | Australia |
| B182 | 3.8 | temp | Japan |
| B045 | 0.0 | temp | Japan |
| B046 | 8.3 | temp | N.A. |
| B047 | 0.0 | adm | Japan |
| B055 | 48.0 | jap | N.A. |
| B057 | 0.0 | temp | N.A. |
| B058 | 23.8 | ind | N.A. |
| B059 | 3.8 | ind | China-Jiangsu |
| B060 | 0.0 | ind | China-Guangdong |
| B061 | 6.1 | ind | China-Guangdong |
| B063 | 0.0 | ind | N.A. |
| B196 | 0.0 | jap | China-Taiwan |
| B197 | 0.6 | ind | China-Taiwan |
| B064 | 0.0 | ind | China-Hunan |
| B065 | 0.0 | ind | N.A. |
| B067 | 0.0 | ind | China-Hebei |
| B070 | 7.2 | temp | China-Heilongjiang |
| B071 | 0.0 | temp | N.A. |
| B072 | 0.0 | ind | China-Anhui |
| B207 | 0.0 | ind | China-Jiangxi |
| B082 | 0.0 | ind | China-Guangxi |
| B083 | 0.0 | ind | China-Hubei |
| B213 | 0.0 | ind | China-Hubei |
| B084 | 3.1 | adm | China-Hubei |
| B085 | 3.1 | ind | China-Hunan |
| B217 | 0.0 | ind | Sichuan |
| B088 | 0.0 | ind | N.A. |
| B091 | 10.1 | ind | China-Yunnan |
| B092 | 0.0 | ind | China-Yunnan |
| B104 | 0.0 | ind | Tibet |
| B226 | 0.0 | temp | China-Ningxia |
| B231 | 0.0 | ind | China-Hubei |
| B112 | 0.0 | ind | China-Guangxi |
| B113 | 0.0 | ind | China-Hunan |
| B114 | 0.0 | ind | Sichuan |
| B115 | 0.0 | ind | Sichuan |
| B116 | 0.0 | ind | Sichuan |
| B119 | 0.0 | ind | China-Guangxi |
| B232 | 1.8 | ind | China-Hunan |
| B233 | 0.0 | ind | China-Hunan |
| B121 | 15.1 | ind | Sichuan |
| B234 | 0.0 | ind | Sichuan |
| B235 | 0.0 | temp | China-Beijing |
| B238 | 0.0 | ind | China-Guangxi |
| B124 | 9.5 | adm | China-Hunan |
| B239 | 17.8 | ind | China-Jiangsu |
| B126 | 0.0 | ind | China-Anhui |
| B240 | 0.0 | temp | China-Henan |
| B128 | 16.8 | ind | N.A. |
| B137 | 0.0 | ind | N.A. |
| B242 | 4.9 | ind | China-Hunan |
| B139 | 4.2 | ind | N.A. |
| B247 | 3.1 | ind | China-Hunan |
| B248 | 0.0 | ind | China-Hunan |
| B150 | 0.0 | ind | N.A. |
| B151 | 0.0 | ind | China-Hunan |
| B153 | 28.1 | ind | N.A. |
| B253 | 6.7 | ind | China-Jiangxi |
| B254 | 19.8 | ind | China-Anhui |
| B154 | 5.0 | temp | N.A. |
| B255 | 0.0 | ind | China-Yunnan |
| B155 | 0.0 | ind | N.A. |
| B156 | 2.0 | ind | Sichuan |
| B157 | 0.0 | ind | N.A. |
| B258 | 0.5 | temp | China-Jilin |
| B259 | 0.0 | ind | China-Anhui |
| B159 | 0.0 | ind | N.A. |
| B160 | 0.0 | temp | N.A. |
| B161 | 2.3 | temp | N.A. |
| CX4 | 1.5 | adm | N.A. |
| CX5 | 6.9 | ind | N.A. |
| CX6 | 38.1 | adm | N.A. |
| CX9 | 0.0 | ind | N.A. |
| CX12 | 0.0 | ind | N.A. |
| CX13 | 0.0 | ind | N.A. |
| CX14 | 0.0 | temp | N.A. |
| CX15 | 46.5 | ind | N.A. |
| CX16 | 0.0 | temp | N.A. |
| CX17 | 0.0 | ind | N.A. |
| CX18 | 0.0 | ind | N.A. |
| CX19 | 0.0 | ind | N.A. |
| CX22 | 10.4 | ind | N.A. |
| CX24 | 0.0 | ind | N.A. |
| CX26 | 0.0 | adm | N.A. |
| CX28 | 35.0 | adm | N.A. |
| CX30 | 7.8 | adm | N.A. |
| CX31 | 0.0 | ind | N.A. |
| CX33 | 0.0 | adm | N.A. |
| CX34 | 0.0 | adm | N.A. |
| CX37 | 0.0 | aus | N.A. |
| CX42 | 0.0 | ind | N.A. |
| CX43 | 2.5 | ind | N.A. |
| CX49 | 0.0 | adm | N.A. |
| CX50 | 0.0 | ind | N.A. |
| CX53 | 0.0 | ind | N.A. |
| CX57 | 12.3 | adm | N.A. |
| CX60 | 0.0 | ind | N.A. |
| CX76 | 0.0 | ind | N.A. |
| CX78 | 10.7 | temp | N.A. |
| CX79 | 0.0 | ind | N.A. |
| CX80 | 0.0 | ind | N.A. |
| CX85 | 11.5 | ind | N.A. |
| CX86 | 0.0 | ind | N.A. |
| CX87 | 0.6 | ind | N.A. |
| CX89 | 0.0 | adm | N.A. |
| CX92 | 9.2 | ind | N.A. |
| CX101 | 0.0 | ind | N.A. |
| CX114 | 0.0 | ind | N.A. |
| CX116 | 9.4 | temp | N.A. |
| CX117 | 0.0 | ind | N.A. |
| CX118 | 4.0 | ind | N.A. |
| CX119 | 1.9 | ind | N.A. |
| CX121 | 18.7 | ind | N.A. |
| CX122 | 27.7 | ind | N.A. |
| CX125 | 4.7 | ind | N.A. |
| CX133 | 0.0 | ind | N.A. |
| CX134 | 15.2 | ind | N.A. |
| CX138 | 0.0 | jap | N.A. |
| CX140 | 0.0 | temp | N.A. |
| CX162 | 0.0 | ind | N.A. |
| CX165 | 6.1 | temp | N.A. |
| CX210 | 9.1 | ind | N.A. |
| CX211 | 0.0 | temp | N.A. |
| CX212 | 0.0 | temp | N.A. |
| CX213 | 23.9 | temp | N.A. |
| CX219 | 1.1 | ind | N.A. |
| CX220 | 6.2 | trop | N.A. |
| CX221 | 5.2 | ind | N.A. |
| CX228 | 0.0 | ind | N.A. |
| CX230 | 0.0 | ind | N.A. |
| CX232 | 0.0 | ind | N.A. |
| CX237 | 4.3 | ind | N.A. |
| CX238 | 9.8 | ind | N.A. |
| CX251 | 0.0 | jap | N.A. |
| CX263 | 0.0 | adm | N.A. |
| CX267 | 0.0 | ind | N.A. |
| CX270 | 0.0 | ind | N.A. |
| CX276 | 4.5 | ind | N.A. |
| CX277 | 6.7 | jap | N.A. |
| CX278 | 0.0 | ind | N.A. |
| CX284 | 0.0 | adm | N.A. |
| CX285 | 0.0 | jap | N.A. |
| CX286 | 0.0 | adm | N.A. |
| CX288 | 26.5 | ind | N.A. |
| CX303 | 5.3 | ind | N.A. |
| CX307 | 24.1 | adm | N.A. |
| CX313 | 0.0 | ind | N.A. |
| CX315 | 4.4 | temp | N.A. |
| CX316 | 34.3 | ind | N.A. |
| CX317 | 7.0 | adm | N.A. |
| CX329 | 4.7 | temp | N.A. |
| CX330 | 3.9 | adm | N.A. |
| CX341 | 6.1 | ind | N.A. |
| CX342 | 5.7 | adm | N.A. |
| CX346 | 0.0 | ind | N.A. |
| CX347 | 10.2 | adm | N.A. |
| CX350 | 4.9 | temp | N.A. |
| CX351 | 0.6 | jap | N.A. |
| CX352 | 4.7 | adm | N.A. |
| CX353 | 0.0 | jap | N.A. |
| CX355 | 0.9 | jap | N.A. |
| CX356 | 6.0 | temp | N.A. |
| CX360 | 0.0 | ind | N.A. |
| CX361 | 14.6 | ind | N.A. |
| CX362 | 0.0 | ind | N.A. |
| CX363 | 0.0 | ind | N.A. |
| CX368 | 5.7 | aus | N.A. |
| CX369 | 20.2 | ind | N.A. |
| CX370 | 20.1 | adm | N.A. |
| CX371 | 1.7 | jap | N.A. |
| CX377 | 0.0 | ind | N.A. |
| CX378 | 1.1 | ind | N.A. |
| CX380 | 0.0 | temp | N.A. |
| CX389 | 0.0 | jap | N.A. |
| CX391 | 0.0 | temp | N.A. |
| CX393 | 2.9 | ind | N.A. |
| CX394 | 1.2 | ind | N.A. |
| CX395 | 0.0 | ind | N.A. |
| CX396 | 0.0 | temp | N.A. |
| CX397 | 16.4 | temp | N.A. |
| CX402 | 2.2 | adm | N.A. |
| CX403 | 0.0 | ind | N.A. |
| CX534 | 0.0 | temp | N.A. |
| CX542 | 0.5 | ind | N.A. |
| CX548 | 0.0 | ind | N.A. |
| IRIS_313-11806 | 4.3 | ind | China |
| IRIS_313-11050 | 0.0 | aus | Bangladesh |
| IRIS_313-11917 | 0.0 | aus | India |
| IRIS_313-10734 | 0.0 | aus | Nepal |
| IRIS_313-11154 | 0.0 | aus | Bangladesh |
| IRIS_313-10833 | 0.0 | ind | India |
| IRIS_313-11557 | 0.0 | ind | Bangladesh |
| IRIS_313-10969 | 0.0 | aus | Brazil |
| IRIS_313-11322 | 0.0 | aus | Bangladesh |
| IRIS_313-11576 | 0.0 | ind | China |
| IRIS_313-11168 | 0.0 | aus | India |
| IRIS_313-11324 | 1.1 | aus | Bangladesh |
| IRIS_313-11456 | 0.0 | aus | India |
| IRIS_313-11170 | 0.0 | aus | India |
| IRIS_313-11868 | 0.0 | ind | China |
| IRIS_313-11171 | 0.0 | aus | India |
| IRIS_313-12275 | 0.0 | ind | China |
| IRIS_313-10930 | 4.0 | aus | Bangladesh |
| IRIS_313-10484 | 0.0 | adm | Philippines |
| IRIS_313-11111 | 0.0 | aus | Bangladesh |
| IRIS_313-11462 | 0.0 | ind | India |
| IRIS_313-11580 | 0.0 | temp | China |
| IRIS_313-11479 | 0.0 | ind | India |
| IRIS_313-11202 | 0.0 | temp | N.A. |
| IRIS_313-10567 | 4.4 | temp | N.A. |
| IRIS_313-11483 | 0.0 | aus | Bangladesh |
| IRIS_313-11890 | 14.0 | temp | Taiwan |
| IRIS_313-10677 | 0.0 | temp | N.A. |
| IRIS_313-11651 | 3.0 | temp | China |
| IRIS_313-10430 | 0.0 | temp | N.A. |
| IRIS_313-10469 | 12.2 | temp | N.A. |
| IRIS_313-11028 | 0.0 | aus | Pakistan |
| IRIS_313-10437 | 0.0 | temp | N.A. |
| IRIS_313-11654 | 0.0 | ind | China |
| IRIS_313-10477 | 2.0 | ind | China |
| IRIS_313-10563 | 0.0 | temp | N.A. |
| IRIS_313-11035 | 0.0 | aus | Pakistan |
| IRIS_313-11153 | 0.0 | jap | India |
| IRIS_313-9449 | 0.0 | aus | Pakistan |
| IRIS_313-10404 | 20.2 | ind | India |
| IRIS_313-9626 | 0.0 | aus | Bangladesh |
| IRIS_313-8342 | 0.0 | aus | Sri Lanka |
| IRIS_313-10423 | 0.0 | jap | Myanmar |
| IRIS_313-9438 | 0.0 | temp | Japan |
| IRIS_313-8890 | 0.0 | temp | Belgium |
| IRIS_313-8655 | 0.0 | aus | Pakistan |
| IRIS_313-8060 | 3.1 | jap | N.A. |
| IRIS_313-8058 | 39.3 | temp | N.A. |
| IRIS_313-8027 | 57.3 | trop | N.A. |
| IRIS_313-8177 | 28.1 | temp | N.A. |
| IRIS_313-8037 | 0.0 | ind | N.A. |
| IRIS_313-8114 | 5.6 | temp | N.A. |
| IRIS_313-8115 | 0.0 | temp | N.A. |
| IRIS_313-8087 | 0.0 | temp | N.A. |
| IRIS_313-7690 | 0.0 | ind | Philippines |
| IRIS_313-7924 | 0.0 | jap | N.A. |
| IRIS_313-8025 | 0.0 | temp | N.A. |
| IRIS_313-8049 | 0.0 | temp | N.A. |
| IRIS_313-8076 | 5.1 | temp | N.A. |
| IRIS_313-8125 | 21.5 | aus | N.A. |
| IRIS_313-8046 | 6.7 | temp | N.A. |
| IRIS_313-8128 | 0.0 | ind | N.A. |
| IRIS_313-8024 | 7.5 | temp | N.A. |
| IRIS_313-8129 | 49.9 | temp | N.A. |
| IRIS_313-8217 | 10.2 | jap | N.A. |
| IRIS_313-8095 | 27.6 | trop | N.A. |
| IRIS_313-9641 | 0.5 | aus | India |
| IRIS_313-8339 | 0.0 | jap | United States |
| IRIS_313-8434 | 2.3 | trop | United States |
| IRIS_313-9066 | 0.0 | ind | Bangladesh |
| IRIS_313-10221 | 0.0 | ind | China |
| IRIS_313-8923 | 0.0 | aro | United States |
| IRIS_313-9703 | 0.0 | temp | Taiwan |
| IRIS_313-10057 | 0.0 | temp | Japan |
| IRIS_313-9790 | 23.0 | temp | Uruguay |
| IRIS_313-9032 | 0.0 | ind | Thailand |
| IRIS_313-9811 | 0.0 | trop | Hungary |
| IRIS_313-8889 | 0.0 | ind | China |
| IRIS_313-9363 | 4.9 | adm | United States |
| IRIS_313-9285 | 0.0 | ind | Myanmar |
| IRIS_313-10234 | 1.8 | ind | Philippines |
| IRIS_313-9937 | 0.0 | aus | Italy |
| IRIS_313-8976 | 0.0 | trop | United States |
| IRIS_313-9081 | 0.0 | trop | Japan |
| IRIS_313-10226 | 0.0 | ind | China |
| IRIS_313-10334 | 4.6 | ind | Indonesia |
| IRIS_313-9184 | 0.0 | ind | China |
| IRIS_313-10373 | 0.0 | trop | Philippines |
| B168 | 0.0 | temp | Japan |
| B171 | 0.0 | temp | Italy |
| B034 | 1.2 | temp | N.A. |
| B036 | 34.4 | temp | N.A. |
| B038 | 0.0 | temp | Egypt |
| B053 | 2.5 | trop | Australia |
| B056 | 0.0 | temp | N.A. |
| B204 | 0.0 | temp | China-Hebei |
| B075 | 0.0 | ind | China-Fujian |
| B228 | 3.7 | temp | China-Yunnan |
| B110 | 0.0 | temp | N.A. |
| B236 | 5.4 | temp | China-Shanxi |
| B147 | 0.0 | ind | China-Hunan |
| B162 | 0.0 | temp | China-Jiangsu |
| B264 | 0.0 | ind | China-Jiangxi |
| CX29 | 0.0 | jap | N.A. |
| CX47 | 0.0 | temp | N.A. |
| CX58 | 0.0 | temp | N.A. |
| CX63 | 0.0 | adm | N.A. |
| CX74 | 15.1 | adm | N.A. |
| CX142 | 6.7 | temp | N.A. |
| CX206 | 0.0 | ind | N.A. |
| CX247 | 2.8 | ind | N.A. |
| CX287 | 0.0 | jap | N.A. |
| CX304 | 0.0 | ind | N.A. |
| CX314 | 42.0 | ind | N.A. |
| CX345 | 0.0 | temp | N.A. |
| CX354 | 5.5 | temp | N.A. |
| CX366 | 0.0 | ind | N.A. |
| IRIS_313-10119 | 12.2 | temp | Italy |
| IRIS_313-10065 | 0.0 | jap | South Korea |
| IRIS_313-11877 | 0.0 | ind | China |
| IRIS_313-11291 | 2.0 | aus | India |
| IRIS_313-11048 | 3.3 | aus | Bangladesh |
| IRIS_313-11049 | 8.9 | aus | Bangladesh |
| IRIS_313-11051 | 0.0 | aus | Bangladesh |
| IRIS_313-11053 | 1.0 | aus | Bangladesh |
| IRIS_313-11061 | 10.7 | aus | Bangladesh |
| IRIS_313-11164 | 10.6 | aus | India |
| IRIS_313-11454 | 0.0 | aus | India |
| IRIS_313-11481 | 7.5 | aus | Bangladesh |
| IRIS_313-11737 | 0.0 | ind | India |
| IRIS_313-10592 | 6.3 | aus | Bangladesh |
| IRIS_313-10602 | 0.0 | aus | Bangladesh |
| IRIS_313-10606 | 1.0 | aus | Bangladesh |
| IRIS_313-10605 | 0.0 | aus | Bangladesh |
| IRIS_313-12053 | 0.0 | ind | Burundi |
| IRIS_313-11748 | 0.0 | ind | China |
| IRIS_313-11863 | 0.0 | ind | China |
| IRIS_313-10504 | 0.0 | ind | China |
| IRIS_313-10966 | 25.1 | ind | Brazil |
| IRIS_313-11930 | 3.8 | ind | Nigeria |
| IRIS_313-11027 | 1.2 | aus | Pakistan |
| IRIS_313-10662 | 0.0 | ind | Sri Lanka |
| IRIS_313-10925 | 5.0 | aus | Nepal |
| IRIS_313-11156 | 1.0 | trop | India |
| IRIS_313-11750 | 0.0 | ind | China |
| IRIS_313-11881 | 0.0 | ind | China |
| IRIS_313-11452 | 0.0 | ind | India |
| IRIS_313-11213 | 2.8 | aus | Bangladesh |
| IRIS_313-11163 | 0.0 | aus | Bangladesh |
| IRIS_313-11738 | 0.0 | trop | India |
| IRIS_313-11871 | 6.0 | ind | China |
| IRIS_313-10608 | 3.2 | aus | India |
| IRIS_313-10429 | 0.0 | temp | N.A. |
| IRIS_313-11884 | 0.0 | ind | China |
| IRIS_313-11872 | 0.0 | ind | China |
| IRIS_313-11619 | 0.0 | aus | India |
| IRIS_313-11374 | 0.0 | aus | India |
| IRIS_313-11753 | 0.0 | adm | China |
| IRIS_313-11910 | 0.0 | ind | China |
| IRIS_313-10564 | 5.4 | temp | N.A. |
| IRIS_313-11595 | 0.0 | ind | India |
| IRIS_313-11020 | 0.0 | aus | Pakistan |
| IRIS_313-10569 | 22.5 | temp | N.A. |
| IRIS_313-10618 | 17.6 | temp | N.A. |
| IRIS_313-10570 | 2.9 | temp | N.A. |
| IRIS_313-12054 | 4.6 | temp | China |
| IRIS_313-11155 | 0.0 | temp | N.A. |
| IRIS_313-11600 | 0.0 | aus | India |
| IRIS_313-10558 | 0.0 | temp | N.A. |
| IRIS_313-11017 | 0.0 | aus | Bangladesh |
| IRIS_313-10453 | 0.7 | temp | N.A. |
| IRIS_313-11809 | 0.0 | ind | Kenya |
| IRIS_313-11829 | 18.9 | ind | Pakistan |
| IRIS_313-11603 | 0.0 | aus | India |
| IRIS_313-11019 | 0.0 | aus | Bangladesh |
| IRIS_313-11232 | 0.0 | aus | India |
| IRIS_313-12050 | 0.0 | ind | Myanmar |
| IRIS_313-11698 | 0.0 | ind | Taiwan |
| IRIS_313-12217 | 0.0 | temp | South Korea |
| IRIS_313-11536 | 0.0 | trop | Georgia |
| IRIS_313-10840 | 0.0 | temp | N.A. |
| IRIS_313-12061 | 0.6 | temp | China |
| IRIS_313-11725 | 0.0 | ind | Japan |
| IRIS_313-15905 | 0.0 | trop | N.A. |
| IRIS_313-8256 | 3.7 | trop | Iran |
| IRIS_313-9002 | 0.0 | temp | Japan |
| IRIS_313-8927 | 1.0 | trop | Taiwan |
| IRIS_313-9523 | 0.0 | jap | Japan |
| IRIS_313-8641 | 0.0 | aus | Bangladesh |
| IRIS_313-8321 | 0.0 | aus | Bangladesh |
| IRIS_313-9610 | 0.0 | aus | India |
| IRIS_313-8789 | 0.0 | aus | Bangladesh |
| IRIS_313-9661 | 0.0 | aus | Bangladesh |
| IRIS_313-8390 | 0.0 | aus | Pakistan |
| IRIS_313-9283 | 0.0 | aus | Pakistan |
| IRIS_313-9880 | 0.0 | trop | Hungary |
| IRIS_313-9346 | 0.0 | temp | Taiwan |
| IRIS_313-9783 | 0.0 | jap | Afghanistan |
| IRIS_313-8062 | 0.0 | temp | N.A. |
| IRIS_313-8069 | 0.0 | temp | N.A. |
| IRIS_313-8168 | 1.1 | adm | N.A. |
| IRIS_313-7641 | 2.6 | ind | N.A. |
| IRIS_313-8084 | 0.0 | trop | N.A. |
| IRIS_313-8112 | 8.9 | temp | N.A. |
| IRIS_313-7665 | 8.7 | ind | N.A. |
| IRIS_313-8066 | 6.4 | temp | N.A. |
| IRIS_313-8074 | 0.0 | temp | N.A. |
| IRIS_313-8041 | 5.7 | temp | N.A. |
| IRIS_313-8029 | 15.3 | adm | N.A. |
| IRIS_313-8118 | 8.0 | temp | N.A. |
| IRIS_313-7698 | 0.0 | ind | N.A. |
| IRIS_313-7907 | 7.6 | trop | N.A. |
| IRIS_313-8068 | 6.2 | temp | N.A. |
| IRIS_313-8216 | 7.6 | ind | N.A. |
| IRIS_313-8138 | 5.1 | temp | N.A. |
| IRIS_313-8143 | 5.4 | ind | N.A. |
| IRIS_313-7778 | 0.0 | ind | N.A. |
| IRIS_313-8145 | 4.6 | temp | N.A. |
| IRIS_313-8149 | 55.4 | temp | N.A. |
| IRIS_313-7797 | 4.9 | adm | N.A. |
| IRIS_313-8167 | 17.4 | ind | N.A. |
| IRIS_313-8400 | 0.0 | trop | Japan |
| IRIS_313-9503 | 8.0 | adm | Philippines |
| IRIS_313-9491 | 5.0 | temp | United States |
| IRIS_313-10071 | 0.0 | jap | Japan |
| IRIS_313-9463 | 21.7 | temp | United States |
| IRIS_313-9410 | 18.9 | temp | United States |
| IRIS_313-9822 | 0.0 | ind | Venezuela |
| IRIS_313-10103 | 0.9 | ind | South Korea |
| IRIS_313-10179 | 0.0 | ind | China |
| IRIS_313-10082 | 3.3 | jap | Japan |
| IRIS_313-9769 | 13.4 | temp | Japan |
| IRIS_313-9468 | 0.0 | temp | China |
| IRIS_313-9839 | 9.7 | temp | Spain |
| IRIS_313-10074 | 18.9 | jap | Japan |
| IRIS_313-9702 | 8.7 | temp | Taiwan |
| IRIS_313-9701 | 0.7 | temp | Taiwan |
| IRIS_313-9838 | 0.0 | temp | New Zealand |
| IRIS_313-10379 | 0.0 | temp | Philippines |
| IRIS_313-10380 | 2.1 | temp | Philippines |
| IRIS_313-10394 | 0.0 | ind | Philippines |
| B018 | 1.3 | trop | Usa |
| B023 | 0.0 | temp | N.A. |
| B024 | 0.7 | ind | Thailand |
| B043 | 0.0 | trop | Australia |
| B183 | 0.0 | temp | Japan |
| B188 | 0.0 | trop | C脭te Divoire |
| B052 | 0.0 | ind | Madagascar |
| B054 | 24.7 | trop | Australia |
| B198 | 0.0 | ind | China-Jiangxi |
| B076 | 0.0 | ind | China-Fujian |
| B208 | 6.0 | ind | China-Fujian |
| B097 | 0.0 | ind | China-Yunnan |
| B221 | 0.0 | aus | China-Yunnan |
| B101 | 0.0 | jap | China-Guizhou |
| B263 | 1.3 | temp | China-Liaoning |
| B163 | 0.0 | ind | China-Anhui |
| B269 | 0.0 | temp | Japan |
| CX32 | 0.0 | jap | N.A. |
| CX56 | 5.8 | temp | N.A. |
| CX109 | 0.0 | jap | N.A. |
| CX124 | 6.4 | ind | N.A. |
| CX128 | 0.0 | ind | N.A. |
| CX130 | 0.0 | ind | N.A. |
| CX131 | 0.0 | ind | N.A. |
| CX139 | 22.6 | jap | N.A. |
| CX214 | 0.0 | adm | N.A. |
| CX306 | 0.0 | temp | N.A. |
| IRIS_313-9782 | 24.9 | temp | Peru |
| IRIS_313-11152 | 8.8 | ind | India |
| IRIS_313-11876 | 0.0 | ind | China |
| IRIS_313-11744 | 0.0 | ind | China |
| IRIS_313-12057 | 0.0 | ind | China |
| IRIS_313-11624 | 0.0 | ind | Nepal |
| IRIS_313-11745 | 0.0 | ind | China |
| IRIS_313-10871 | 9.3 | aus | India |
| IRIS_313-10875 | 3.7 | aus | India |
| IRIS_313-10852 | 0.0 | aus | India |
| IRIS_313-11054 | 0.0 | aus | Bangladesh |
| IRIS_313-11055 | 1.1 | aus | Bangladesh |
| IRIS_313-11057 | 0.0 | aus | Bangladesh |
| IRIS_313-11166 | 0.0 | aus | India |
| IRIS_313-11013 | 0.6 | aus | Bangladesh |
| IRIS_313-12210 | 0.0 | ind | Vietnam |
| IRIS_313-10450 | 0.0 | ind | China |
| IRIS_313-11855 | 0.0 | ind | China |
| IRIS_313-11858 | 1.6 | ind | China |
| IRIS_313-10593 | 0.0 | aus | Bangladesh |
| IRIS_313-10595 | 0.0 | aus | Bangladesh |
| IRIS_313-10544 | 0.0 | ind | India |
| IRIS_313-10721 | 10.2 | ind | Sri Lanka |
| IRIS_313-11909 | 0.0 | ind | China |
| IRIS_313-11943 | 0.0 | ind | Nepal |
| IRIS_313-10968 | 0.0 | ind | Brazil |
| IRIS_313-11323 | 0.0 | aus | Bangladesh |
| IRIS_313-11888 | 0.0 | aus | Pakistan |
| IRIS_313-12055 | 0.0 | aus | Bangladesh |
| IRIS_313-10735 | 0.0 | aus | Nepal |
| IRIS_313-11577 | 0.0 | temp | China |
| IRIS_313-12236 | 0.0 | ind | China |
| IRIS_313-11629 | 1.5 | aro | Nepal |
| IRIS_313-11985 | 0.0 | ind | China |
| IRIS_313-11018 | 0.0 | aus | Bangladesh |
| IRIS_313-12139 | 0.0 | aus | Nepal |
| IRIS_313-11951 | 0.0 | ind | China |
| IRIS_313-10716 | 0.0 | ind | Sri Lanka |
| IRIS_313-10561 | 0.0 | ind | China |
| IRIS_313-10476 | 0.0 | ind | Sri Lanka |
| IRIS_313-11982 | 0.0 | aus | Bangladesh |
| IRIS_313-10964 | 0.0 | aus | Bangladesh |
| IRIS_313-10631 | 0.0 | temp | N.A. |
| IRIS_313-11655 | 2.6 | ind | China |
| IRIS_313-11174 | 0.0 | aus | India |
| IRIS_313-11175 | 0.0 | aus | India |
| IRIS_313-10965 | 0.0 | aus | Bangladesh |
| IRIS_313-10502 | 0.0 | ind | China |
| IRIS_313-12237 | 0.0 | ind | China |
| IRIS_313-11885 | 0.6 | ind | China |
| IRIS_313-10888 | 0.0 | trop | N.A. |
| IRIS_313-11047 | 0.0 | aus | Bangladesh |
| IRIS_313-11071 | 15.9 | ind | Laos |
| IRIS_313-10708 | 2.3 | trop | United States |
| IRIS_313-11571 | 0.0 | ind | China |
| IRIS_313-12186 | 2.2 | ind | Laos |
| IRIS_313-10827 | 0.0 | trop | Philippines |
| IRIS_313-12329 | 0.0 | ind | Laos |
| IRIS_313-10598 | 0.0 | aus | Bangladesh |
| IRIS_313-11016 | 0.0 | aus | Bangladesh |
| IRIS_313-11578 | 0.7 | ind | China |
| IRIS_313-11441 | 7.6 | ind | Philippines |
| IRIS_313-11766 | 0.0 | ind | Madagascar |
| IRIS_313-10585 | 0.0 | adm | Japan |
| IRIS_313-11617 | 0.9 | aus | India |
| IRIS_313-11582 | 0.0 | temp | China |
| IRIS_313-9555 | 0.0 | ind | China |
| IRIS_313-9137 | 0.0 | aus | India |
| IRIS_313-8554 | 0.0 | aus | India |
| IRIS_313-9368 | 1.0 | aus | Bangladesh |
| IRIS_313-8134 | 30.8 | trop | N.A. |
| IRIS_313-8136 | 21.6 | temp | N.A. |
| IRIS_313-8137 | 34.5 | ind | N.A. |
| IRIS_313-8063 | 20.4 | ind | N.A. |
| IRIS_313-8166 | 0.0 | temp | N.A. |
| IRIS_313-8111 | 48.7 | temp | N.A. |
| IRIS_313-8158 | 0.0 | temp | N.A. |
| IRIS_313-7870 | 1.9 | trop | N.A. |
| IRIS_313-8073 | 0.0 | trop | N.A. |
| IRIS_313-8154 | 7.6 | adm | N.A. |
| IRIS_313-8003 | 7.9 | jap | N.A. |
| IRIS_313-7902 | 10.1 | trop | N.A. |
| IRIS_313-8121 | 6.0 | temp | N.A. |
| IRIS_313-8124 | 21.0 | trop | N.A. |
| IRIS_313-8023 | 60.3 | temp | N.A. |
| IRIS_313-8205 | 1.3 | temp | N.A. |
| IRIS_313-8141 | 10.6 | temp | N.A. |
| IRIS_313-8151 | 0.0 | temp | N.A. |
| IRIS_313-8660 | 0.0 | ind | Sri Lanka |
| IRIS_313-9774 | 0.0 | temp | Turkey |
| IRIS_313-9140 | 0.0 | adm | China |
| IRIS_313-10211 | 0.0 | ind | China |
| IRIS_313-9730 | 0.0 | ind | China |
| IRIS_313-9233 | 14.2 | temp | China |
| IRIS_313-9193 | 3.5 | temp | Brazil |
| IRIS_313-9884 | 8.5 | temp | Japan |
| IRIS_313-9778 | 0.0 | ind | Argentina |
| IRIS_313-9974 | 0.0 | temp | South Korea |
| IRIS_313-10080 | 0.0 | jap | Japan |
| IRIS_313-9379 | 0.0 | temp | South Korea |
| IRIS_313-10067 | 0.0 | jap | South Korea |
| IRIS_313-10096 | 13.3 | trop | South Korea |
| IRIS_313-10097 | 0.0 | temp | South Korea |
| IRIS_313-9995 | 0.0 | temp | South Korea |
| IRIS_313-9970 | 0.0 | ind | Sri Lanka |
| IRIS_313-10075 | 0.0 | ind | Japan |
| IRIS_313-10014 | 33.5 | temp | Italy |
| IRIS_313-9706 | 8.1 | ind | Taiwan |
| IRIS_313-9708 | 2.2 | ind | Taiwan |
| IRIS_313-9388 | 0.0 | ind | Madagascar |
| IRIS_313-9550 | 0.0 | trop | United States |
| IRIS_313-10078 | 0.0 | jap | Japan |
| IRIS_313-8645 | 0.0 | ind | China |
| IRIS_313-8380 | 0.0 | aus | China |
| IRIS_313-9771 | 0.0 | trop | Austria |
| B011 | 0.0 | ind | India |
| B014 | 24.8 | jap | N.A. |
| B190 | 0.0 | trop | Nigeria |
| B062 | 0.0 | ind | China-Jiangxi |
| B199 | 0.0 | temp | China-Jiangxi |
| B066 | 0.0 | temp | N.A. |
| B203 | 0.0 | ind | China-Yunnan |
| B077 | 0.0 | temp | N.A. |
| B081 | 0.0 | ind | China-Guangdong |
| B214 | 0.0 | ind | China-Hubei |
| B215 | 0.0 | temp | China-Hunan |
| B216 | 0.0 | ind | China-Henan |
| B087 | 0.0 | ind | Sichuan |
| B106 | 0.0 | ind | China-Yunnan |
| B130 | 0.0 | ind | China-Hunan |
| CX11 | 0.0 | adm | N.A. |
| CX54 | 0.0 | ind | N.A. |
| CX65 | 0.0 | jap | N.A. |
| CX88 | 0.0 | ind | N.A. |
| CX110 | 17.3 | aro | N.A. |
| CX112 | 0.0 | aro | N.A. |
| CX233 | 0.0 | ind | N.A. |
| CX236 | 0.0 | adm | N.A. |
| CX241 | 0.0 | jap | N.A. |
| CX248 | 1.0 | adm | N.A. |
| CX269 | 0.0 | trop | N.A. |
| CX367 | 0.0 | adm | N.A. |
| CX383 | 8.9 | temp | N.A. |
| CX384 | 0.6 | jap | N.A. |
| IRIS_313-8232 | 4.6 | ind | Philippines |
| IRIS_313-9800 | 4.2 | trop | Japan |
| IRIS_313-9389 | 0.0 | trop | Philippines |
| IRIS_313-10876 | 4.5 | aus | India |
| IRIS_313-10877 | 6.3 | aus | India |
| IRIS_313-10878 | 13.6 | aus | India |
| IRIS_313-10879 | 0.0 | aus | India |
| IRIS_313-10892 | 0.0 | aus | India |
| IRIS_313-11298 | 6.0 | aus | India |
| IRIS_313-11272 | 4.6 | aus | India |
| IRIS_313-11274 | 0.0 | aus | India |
| IRIS_313-11255 | 0.0 | ind | India |
| IRIS_313-10924 | 0.0 | ind | Nepal |
| IRIS_313-11052 | 0.6 | aus | Bangladesh |
| IRIS_313-11656 | 15.8 | ind | Indonesia |
| IRIS_313-12070 | 1.8 | trop | Malaysia |
| IRIS_313-11878 | 0.0 | ind | China |
| IRIS_313-11423 | 0.0 | ind | Philippines |
| IRIS_313-11379 | 0.0 | trop | Philippines |
| IRIS_313-11856 | 0.0 | ind | China |
| IRIS_313-11857 | 0.0 | ind | China |
| IRIS_313-11859 | 0.0 | ind | China |
| IRIS_313-11411 | 0.0 | ind | Brazil |
| IRIS_313-11796 | 0.0 | trop | China |
| IRIS_313-11573 | 0.0 | temp | China |
| IRIS_313-11929 | 0.0 | trop | Philippines |
| IRIS_313-11860 | 0.0 | ind | China |
| IRIS_313-11861 | 0.0 | ind | China |
| IRIS_313-11575 | 0.0 | ind | China |
| IRIS_313-11948 | 0.0 | ind | China |
| IRIS_313-11695 | 0.0 | aro | Taiwan |
| IRIS_313-11537 | 0.0 | ind | Philippines |
| IRIS_313-11489 | 7.1 | ind | India |
| IRIS_313-12094 | 3.6 | aro | Bangladesh |
| IRIS_313-10963 | 0.0 | aus | Bangladesh |
| IRIS_313-11867 | 0.0 | ind | China |
| IRIS_313-11919 | 11.4 | ind | India |
| IRIS_313-12321 | 5.1 | trop | Laos |
| IRIS_313-11949 | 0.0 | ind | China |
| IRIS_313-11882 | 0.0 | ind | China |
| IRIS_313-11803 | 0.0 | ind | China |
| IRIS_313-10458 | 0.0 | ind | China |
| IRIS_313-12164 | 0.0 | trop | Cambodia |
| IRIS_313-10835 | 0.0 | ind | India |
| IRIS_313-11952 | 0.0 | ind | China |
| IRIS_313-11172 | 0.0 | aus | India |
| IRIS_313-11173 | 0.0 | aus | India |
| IRIS_313-11436 | 0.0 | ind | Ivory Coast |
| IRIS_313-11579 | 2.0 | ind | China |
| IRIS_313-10515 | 0.0 | ind | Taiwan |
| IRIS_313-11157 | 0.0 | ind | Taiwan |
| IRIS_313-11620 | 0.0 | ind | India |
| IRIS_313-11618 | 0.0 | aus | India |
| IRIS_313-11790 | 0.0 | ind | Madagascar |
| IRIS_313-12138 | 0.0 | ind | Bhutan |
| IRIS_313-12012 | 0.0 | ind | China |
| IRIS_313-10865 | 0.0 | trop | N.A. |
| IRIS_313-11296 | 0.0 | ind | India |
| IRIS_313-11425 | 7.2 | adm | Brazil |
| IRIS_313-10614 | 0.0 | ind | China-Hong Kong |
| IRIS_313-10718 | 13.0 | aus | Sri Lanka |
| IRIS_313-11362 | 0.0 | aro | India |
| IRIS_313-11037 | 0.0 | aus | Pakistan |
| IRIS_313-11495 | 0.0 | jap | Indonesia |
| IRIS_313-10884 | 0.0 | trop | N.A. |
| IRIS_313-10897 | 23.1 | ind | India |
| IRIS_313-11427 | 0.0 | trop | Brazil |
| IRIS_313-10991 | 0.0 | trop | Philippines |
| IRIS_313-10507 | 0.0 | jap | N.A. |
| IRIS_313-10967 | 0.0 | temp | Brazil |
| IRIS_313-10828 | 0.0 | trop | Philippines |
| IRIS_313-10829 | 0.0 | trop | Philippines |
| IRIS_313-11956 | 0.0 | adm | Nepal |
| IRIS_313-10946 | 0.0 | trop | Indonesia |
| IRIS_313-11435 | 0.0 | ind | Ivory Coast |
| IRIS_313-10752 | 0.0 | trop | United States |
| IRIS_313-10703 | 0.0 | trop | Malaysia |
| IRIS_313-11004 | 8.2 | trop | Indonesia |
| IRIS_313-11189 | 13.8 | adm | Ussr |
| IRIS_313-11539 | 5.3 | trop | N.A. |
| IRIS_313-12311 | 0.0 | trop | Laos |
| IRIS_313-10600 | 0.9 | aus | Bangladesh |
| IRIS_313-8747 | 11.6 | aro | Iran |
| IRIS_313-8410 | 0.0 | aus | Bangladesh |
| IRIS_313-7856 | 0.0 | trop | N.A. |
| IRIS_313-7863 | 0.0 | ind | N.A. |
| IRIS_313-8159 | 0.0 | temp | N.A. |
| IRIS_313-8116 | 15.4 | temp | N.A. |
| IRIS_313-8010 | 0.0 | trop | N.A. |
| IRIS_313-7909 | 0.6 | trop | N.A. |
| IRIS_313-7728 | 0.0 | ind | N.A. |
| IRIS_313-8123 | 17.6 | temp | N.A. |
| IRIS_313-8140 | 21.1 | trop | N.A. |
| IRIS_313-8142 | 18.2 | temp | N.A. |
| IRIS_313-7720 | 0.0 | ind | N.A. |
| IRIS_313-8647 | 0.0 | ind | India |
| IRIS_313-8986 | 0.0 | ind | India |
| IRIS_313-9176 | 0.0 | trop | India |
| IRIS_313-9452 | 15.2 | trop | United States |
| IRIS_313-8960 | 0.0 | ind | United States |
| IRIS_313-9267 | 0.0 | trop | Venezuela |
| IRIS_313-9529 | 0.0 | trop | Bhutan |
| IRIS_313-10190 | 0.0 | trop | China |
| IRIS_313-10061 | 34.9 | jap | Portugal |
| IRIS_313-10114 | 0.0 | ind | Burundi |
| IRIS_313-9980 | 32.0 | jap | Argentina |
| IRIS_313-10094 | 0.0 | temp | South Korea |
| IRIS_313-10154 | 2.2 | ind | China |
| IRIS_313-9887 | 0.0 | temp | South Korea |
| IRIS_313-10025 | 0.0 | temp | Madagascar |
| IRIS_313-9924 | 0.0 | ind | South Korea |
| IRIS_313-9759 | 65.2 | temp | Portugal |
| IRIS_313-10076 | 12.3 | jap | Japan |
| IRIS_313-9976 | 0.0 | ind | South Korea |
| IRIS_313-10077 | 0.0 | jap | Japan |
| IRIS_313-9890 | 0.0 | temp | South Korea |
| IRIS_313-9611 | 0.0 | ind | India |
| IRIS_313-9372 | 0.0 | ind | China |
| IRIS_313-10337 | 0.0 | ind | Indonesia |
| IRIS_313-8731 | 0.0 | ind | India |
| IRIS_313-8778 | 0.0 | trop | United States |
| IRIS_313-9723 | 0.0 | ind | China |
| IRIS_313-10062 | 0.0 | jap | Greece |
| IRIS_313-8481 | 4.9 | temp | China |
| IRIS_313-10150 | 0.0 | ind | India |
| B068 | 0.0 | temp | N.A. |
| B205 | 0.0 | temp | China-Jiangsu |
| B079 | 0.0 | ind | China-Guangdong |
| B086 | 0.0 | adm | China-Hunan |
| B089 | 0.0 | ind | China-Yunnan |
| B094 | 0.0 | ind | China-Yunnan |
| B095 | 0.0 | ind | China-Yunnan |
| B246 | 44.9 | ind | China-Yunnan |
| B260 | 0.0 | ind | Sichuan |
| B261 | 0.0 | ind | Sichuan |
| B266 | 0.0 | trop | China-Yunnan |
| B164 | 0.0 | aus | China-Yunnan |
| CX55 | 0.0 | ind | N.A. |
| CX104 | 0.0 | aro | N.A. |
| CX123 | 0.0 | ind | N.A. |
| CX242 | 0.0 | adm | N.A. |
| CX262 | 0.0 | jap | N.A. |
| IRIS_313-10026 | 0.0 | ind | Madagascar |
| IRIS_313-8694 | 0.0 | trop | Brazil |
| IRIS_313-10327 | 0.0 | temp | Peru |
| IRIS_313-10671 | 18.1 | ind | India |
| IRIS_313-10861 | 0.0 | aus | India |
| IRIS_313-11295 | 13.0 | adm | India |
| IRIS_313-11304 | 0.0 | ind | India |
| IRIS_313-11852 | 0.0 | ind | China |
| IRIS_313-10619 | 0.0 | trop | N.A. |
| IRIS_313-11854 | 0.0 | ind | China |
| IRIS_313-10594 | 0.0 | aus | Bangladesh |
| IRIS_313-12337 | 0.0 | trop | Laos |
| IRIS_313-11066 | 0.0 | aro | Bangladesh |
| IRIS_313-11862 | 0.0 | ind | China |
| IRIS_313-11802 | 0.0 | temp | China |
| IRIS_313-11599 | 0.0 | ind | India |
| IRIS_313-11414 | 0.0 | ind | India |
| IRIS_313-10999 | 0.0 | trop | Indonesia |
| IRIS_313-12002 | 0.0 | aus | Bangladesh |
| IRIS_313-12348 | 0.0 | trop | Laos |
| IRIS_313-10748 | 0.0 | ind | Vietnam |
| IRIS_313-12272 | 0.0 | trop | Philippines |
| IRIS_313-11736 | 0.0 | aus | Philippines |
| IRIS_313-12352 | 0.0 | trop | N.A. |
| IRIS_313-11445 | 0.0 | ind | India |
| IRIS_313-12226 | 13.4 | trop | Laos |
| IRIS_313-10992 | 0.0 | trop | Philippines |
| IRIS_313-11966 | 0.0 | ind | China |
| IRIS_313-11953 | 0.0 | ind | China |
| IRIS_313-11568 | 0.0 | ind | Nepal |
| IRIS_313-12183 | 0.0 | aus | Nepal |
| IRIS_313-10560 | 0.0 | ind | China |
| IRIS_313-10836 | 0.0 | ind | India |
| IRIS_313-11805 | 0.0 | ind | China |
| IRIS_313-12010 | 0.0 | ind | China |
| IRIS_313-11911 | 0.0 | ind | China |
| IRIS_313-11210 | 0.0 | aus | Bangladesh |
| IRIS_313-10859 | 0.0 | ind | India |
| IRIS_313-11934 | 0.0 | ind | Philippines |
| IRIS_313-10552 | 0.0 | trop | United States |
| IRIS_313-10583 | 0.0 | temp | N.A. |
| IRIS_313-11673 | 0.0 | ind | Philippines |
| IRIS_313-11918 | 0.0 | ind | India |
| IRIS_313-12265 | 0.0 | trop | Laos |
| IRIS_313-12059 | 0.0 | temp | China |
| IRIS_313-10796 | 0.0 | trop | Indonesia |
| IRIS_313-11954 | 0.0 | ind | China |
| IRIS_313-7850 | 0.0 | ind | N.A. |
| IRIS_313-8132 | 10.5 | temp | N.A. |
| IRIS_313-7769 | 0.0 | ind | N.A. |
| IRIS_313-8139 | 11.0 | temp | N.A. |
| IRIS_313-8387 | 6.0 | trop | China |
| IRIS_313-8454 | 0.0 | ind | Taiwan |
| IRIS_313-9609 | 0.0 | ind | India |
| IRIS_313-8982 | 18.6 | ind | India |
| IRIS_313-9572 | 0.0 | ind | Bhutan |
| IRIS_313-10079 | 0.0 | jap | Japan |
| IRIS_313-10189 | 0.0 | ind | China |
| IRIS_313-10164 | 0.0 | aus | China |
| IRIS_313-9366 | 15.3 | trop | United States |
| IRIS_313-9698 | 2.4 | temp | North Korea |
| IRIS_313-9351 | 0.0 | aus | India |
| IRIS_313-10129 | 0.0 | ind | China |
| IRIS_313-9705 | 0.0 | ind | Taiwan |
| IRIS_313-8302 | 0.0 | temp | Bhutan |
| IRIS_313-9568 | 0.0 | trop | Malaysia |
| B184 | 0.0 | ind | Vietnam |
| B049 | 0.0 | aus | Nepal |
| B219 | 8.9 | ind | China-Yunnan |
| B109 | 1.7 | temp | N.A. |
| B134 | 4.9 | trop | N.A. |
| B241 | 0.0 | trop | China-Yunnan |
| B244 | 3.2 | ind | China-Henan |
| CX66 | 16.0 | adm | N.A. |
| CX102 | 3.3 | ind | N.A. |
| CX227 | 0.0 | ind | N.A. |
| IRIS_313-8486 | 0.8 | trop | Bhutan |
| IRIS_313-9745 | 0.5 | jap | Madagascar |
| IRIS_313-10854 | 5.0 | aus | India |
| IRIS_313-10857 | 6.1 | ind | India |
| IRIS_313-10676 | 3.7 | ind | India |
| IRIS_313-10862 | 0.0 | trop | N.A. |
| IRIS_313-10869 | 0.0 | aus | India |
| IRIS_313-10882 | 0.0 | aus | India |
| IRIS_313-10891 | 0.0 | aus | India |
| IRIS_313-10895 | 0.0 | trop | N.A. |
| IRIS_313-11306 | 10.0 | ind | India |
| IRIS_313-11062 | 0.0 | aro | Bangladesh |
| IRIS_313-11515 | 0.0 | ind | N.A. |
| IRIS_313-11070 | 0.0 | aro | Bangladesh |
| IRIS_313-11692 | 2.4 | ind | Taiwan |
| IRIS_313-10444 | 1.1 | adm | Central America |
| IRIS_313-10503 | 3.3 | ind | China |
| IRIS_313-11067 | 0.0 | aus | Bangladesh |
| IRIS_313-10452 | 11.0 | ind | China |
| IRIS_313-11977 | 0.0 | ind | Madagascar |
| IRIS_313-11869 | 2.9 | ind | China |
| IRIS_313-10799 | 0.0 | trop | Indonesia |
| IRIS_313-12009 | 0.0 | ind | China |
| IRIS_313-11347 | 5.5 | ind | Philippines |
| IRIS_313-11955 | 0.0 | ind | China |
| IRIS_313-12065 | 0.0 | aus | Nigeria |
| IRIS_313-11069 | 0.0 | aro | Bangladesh |
| IRIS_313-10866 | 3.3 | trop | N.A. |
| IRIS_313-10867 | 19.5 | trop | N.A. |
| IRIS_313-10644 | 1.0 | trop | Philippines |
| IRIS_313-12258 | 10.8 | trop | Laos |
| IRIS_313-10834 | 0.0 | trop | N.A. |
| IRIS_313-12180 | 0.0 | ind | Nepal |
| IRIS_313-11031 | 0.0 | aus | Pakistan |
| IRIS_313-11068 | 0.0 | aro | Bangladesh |
| IRIS_313-10959 | 2.0 | trop | Indonesia |
| IRIS_313-9404 | 0.0 | adm | Bhutan |
| IRIS_313-7933 | 0.0 | trop | Nepal |
| IRIS_313-8147 | 13.5 | ind | N.A. |
| IRIS_313-7994 | 0.0 | jap | N.A. |
| IRIS_313-7646 | 0.0 | trop | N.A. |
| IRIS_313-8876 | 0.0 | trop | France |
| IRIS_313-10224 | 0.0 | ind | China |
| IRIS_313-9758 | 0.0 | aus | Taiwan |
| IRIS_313-8857 | 0.0 | trop | Bhutan |
| IRIS_313-9732 | 0.0 | ind | Madagascar |
| IRIS_313-9375 | 14.2 | jap | Malaysia |
| IRIS_313-9469 | 0.0 | ind | China |
| IRIS_313-9048 | 0.0 | temp | Bhutan |
| IRIS_313-9519 | 0.0 | trop | Suriname |
| IRIS_313-8745 | 0.0 | ind | Haiti |
| IRIS_313-8637 | 0.0 | trop | Bhutan |
| IRIS_313-8627 | 0.0 | temp | United States |
| B189 | 0.0 | trop | C脭te Divoire |
| B100 | 0.0 | temp | N.A. |
| CX141 | 6.5 | ind | N.A. |
| IRIS_313-7722 | 0.0 | ind | N.A. |
| IRIS_313-10894 | 0.0 | aus | India |
| IRIS_313-10935 | 0.0 | ind | Indonesia |
| IRIS_313-11383 | 0.0 | ind | Vietnam |
| IRIS_313-12332 | 0.0 | trop | Laos |
| IRIS_313-12033 | 15.2 | ind | China |
| IRIS_313-10783 | 12.1 | jap | Indonesia |
| IRIS_313-12350 | 0.0 | trop | Laos |
| IRIS_313-11950 | 0.0 | ind | China |
| IRIS_313-12336 | 6.3 | trop | Laos |
| IRIS_313-11908 | 0.0 | temp | China |
| IRIS_313-10832 | 0.0 | trop | Philippines |
| IRIS_313-10743 | 0.0 | trop | Indonesia |
| IRIS_313-11691 | 0.0 | ind | Bhutan |
| IRIS_313-11792 | 0.0 | ind | Madagascar |
| IRIS_313-9101 | 0.0 | jap | Malaysia |
| IRIS_313-8815 | 4.5 | trop | Bhutan |
| IRIS_313-10176 | 0.0 | trop | Thailand |
| IRIS_313-9605 | 0.0 | ind | India |
| IRIS_313-8754 | 0.0 | ind | India |
| B212 | 0.0 | temp | China-Guangdong |
| B218 | 0.0 | temp | China-Yunnan |
| B223 | 0.0 | temp | China-Guizhou |
| B103 | 0.0 | temp | N.A. |
| B245 | 20.1 | trop | Sichuan |
| IRIS_313-11384 | 0.0 | ind | Vietnam |
| IRIS_313-12031 | 0.0 | trop | Philippines |
| IRIS_313-7885 | 0.0 | trop | Indonesia |
| IRIS_313-7959 | 0.0 | trop | N.A. |
| IRIS_313-9197 | 0.0 | temp | India |
| B111 | 0.0 | temp | N.A. |

^1)^ The accession ID can be reached in the database (<http://www.rmbreeding.cn/3ksnp> ) for further details;

^2)^ temp = temperate *japonica*, trop = tropical *japonica*, ind = *indica*, aus = Aus type, adm = admixture, jap = *japonica*, aro = Aroma.
